# Supplementary material for: Nanoparticles targeting mutant p53 overcome chemoresistance and tumor recurrence in non-small cell lung cancer
Source: Nat Commun. 2024 Mar 29;15:2759. doi: 10.1038/s41467-024-47080-3 (PMC10980692; doi:10.1038/s41467-024-47080-3)
Supplement: Supplementary file 1 — Supplementary information [file 41467_2024_47080_MOESM1_ESM.pdf]

Supplementary Information for

Nanoparticles targeting mutant p53 overcome chemoresistance and tumor recurrence in non-small cell lung cancer

Yu-Yang Bi[1], Qiu Chen[1], Ming-Yuan Yang[1], Lei Xing[1,2], Hu-Lin Jiang[1,2,3]*

[1]State Key Laboratory of Natural Medicines, China Pharmaceutical University, Nanjing 210009, China. [2]Jiangsu Key Laboratory of Druggability of Biopharmaceuticals, China Pharmaceutical University, Nanjing 210009, China. [3]College of Pharmacy, Yanbian University, No.977, Gongyan Road, Yanji 133000, China.
email: jianghulin3@gmail.com

Supplementary figures

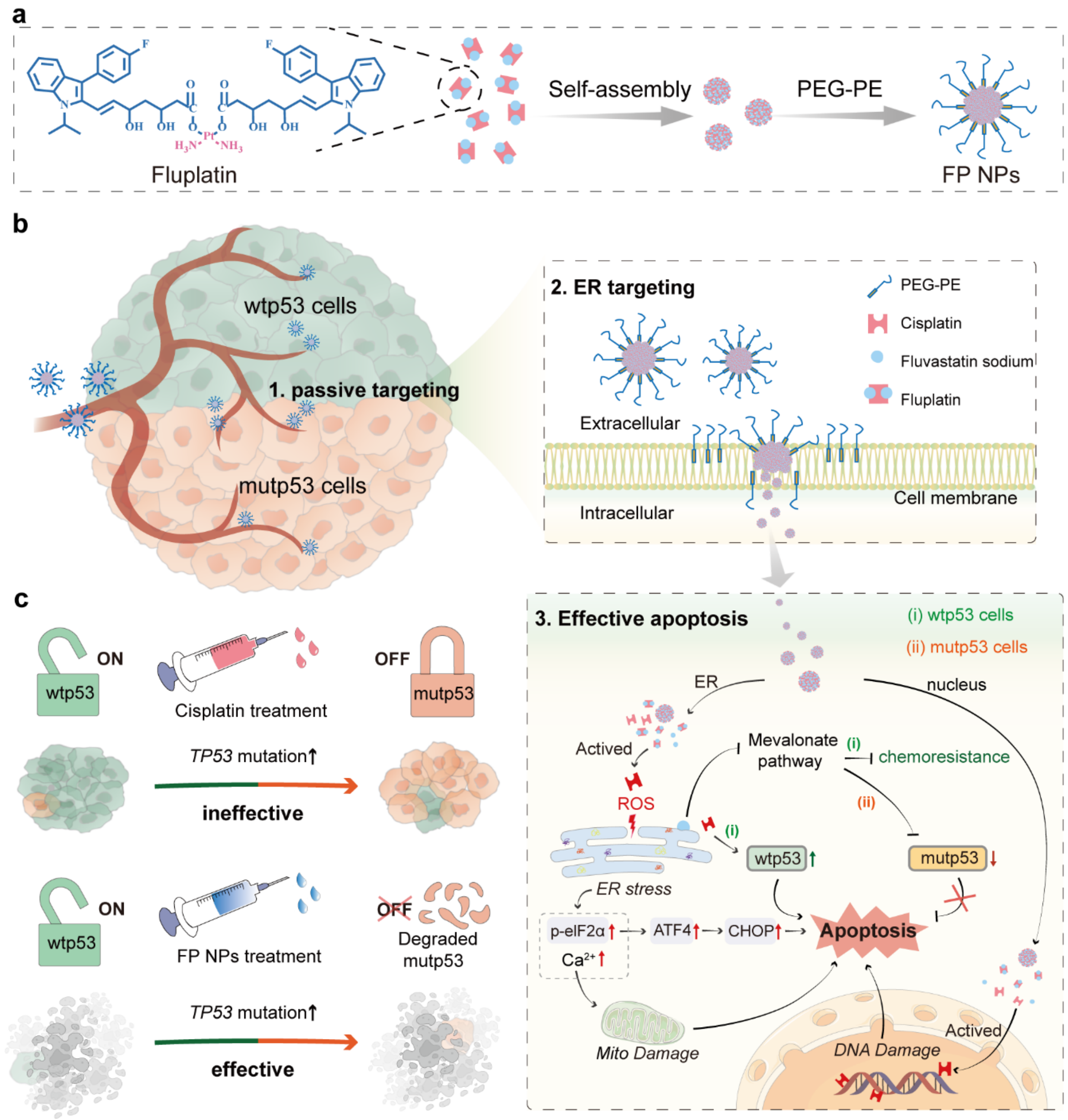

Supplementary Fig. 1 Study summary. **a** Chemical structure of Fluplatin and schematic diagram of the preparation of the FP NPs. **b** FP NPs can be passively targeted to tumor sites (i), Fluplatin self-assembled nanoparticles accumulate more in the endoplasmic reticulum by PEG-PE insertion into cell membranes (ii). FP NPs enhance apoptosis by increasing ERS in wtp53 cells; in mutp53 cells, FP NPs break the inhibition of ERS and DNA damage-induced apoptosis via specific degradation of mutp53, thus exerting an efficient antitumor effect (iii). **c** Schematic illustration of treatment with cisplatin and FP NPs in mutp53 or wtp53 cells. Created with BioRender.com.

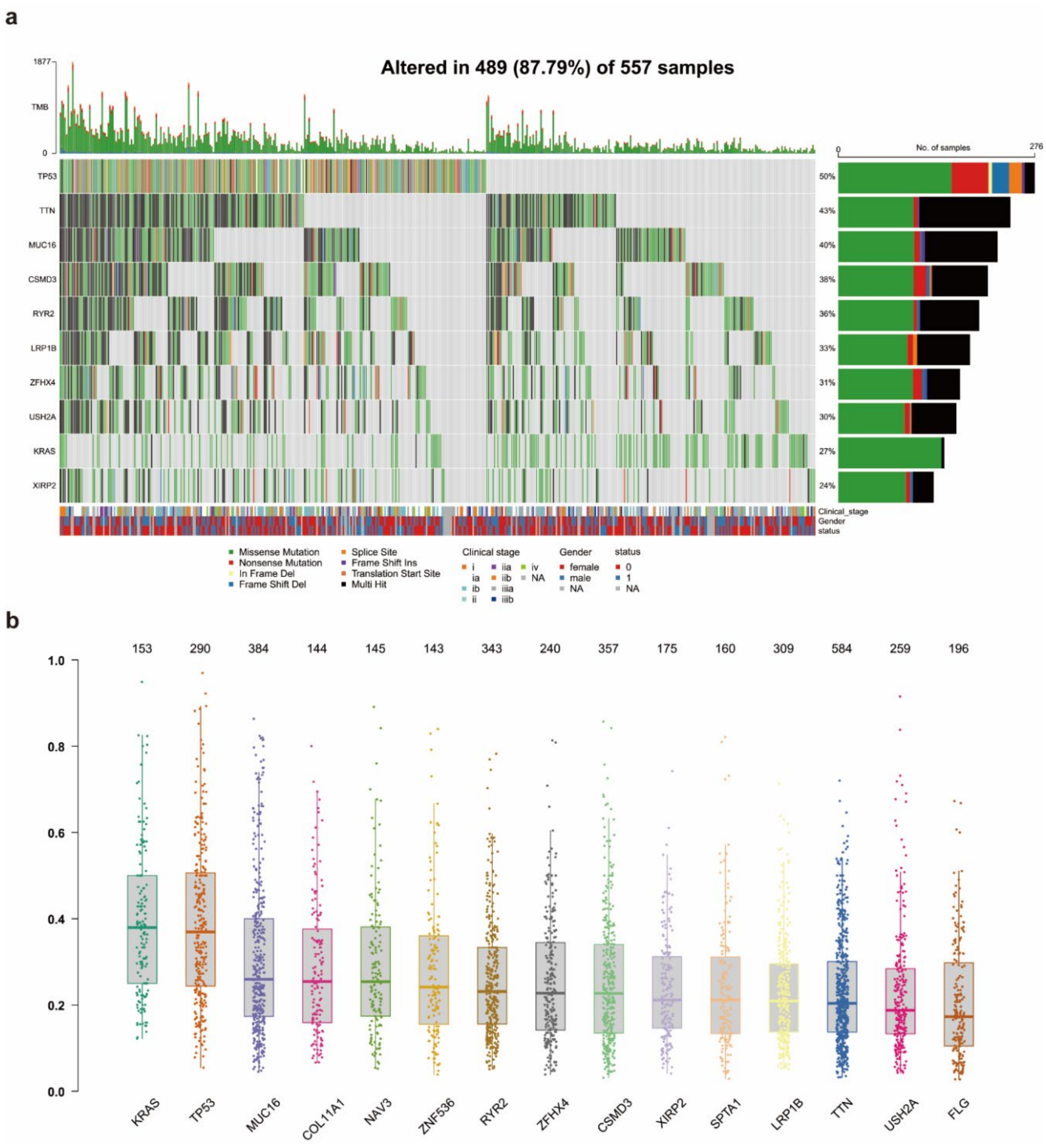

Supplementary Fig. 2 Analyses of gene mutations in LUAD samples. a Waterfall plot of detailed mutation information of the top 10 genes in each sample in the LUAD TCGA cohorts, with various color annotations to distinguish different mutation types, clinical stages, and genders. **b** Variant allele frequency in each sample.

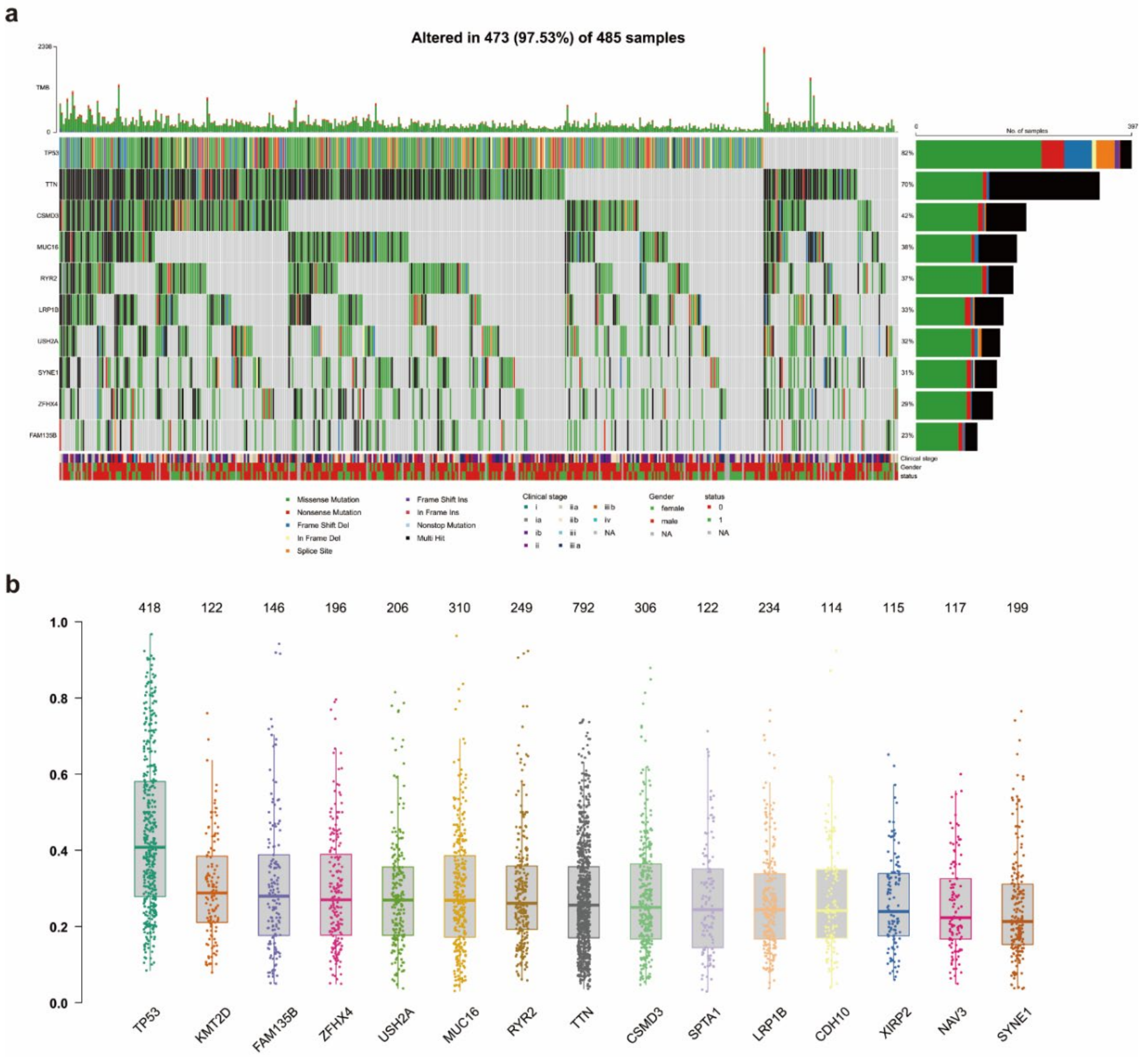

Supplementary Fig. 3 Analyses of gene mutations in LUSC samples. a Waterfall plot of detailed mutation information of the top 10 genes in each sample in the LUSC TCGA cohorts, with various color annotations to distinguish different mutation types, clinical stages, and genders. **b** Variant allele frequency in each sample.

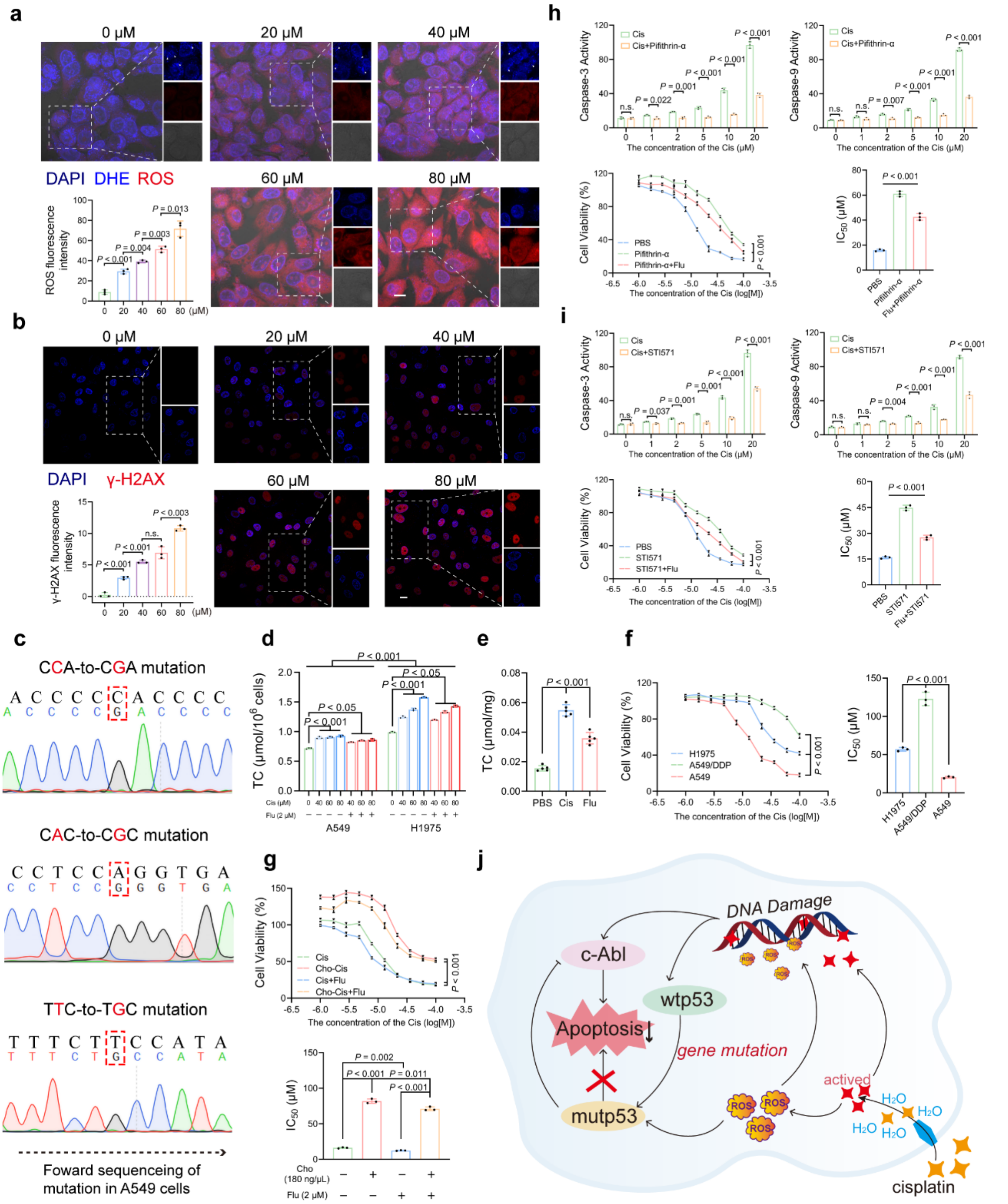

Supplementary Fig. 4 Mechanistic validation of a vicious cycle between cisplatin and p53. Confocal images of ROS (**a**) and γ-H2AX (**b**) in A549 cells after 12 h of treatment with different concentrations of cisplatin and quantification of their fluorescence intensity (n = 3 independent samples; one-way ANOVA followed by Tukey's HSD post hoc test). Scale bars, 10 μm. **c** Forward sequencing of mutations in A549 cells treated with cisplatin (low dose) for 1 month. **d** Intracellular TC levels in A549 and H1975 cells after 12 h of treatment with different concentrations of cisplatin (n = 3 independent samples; one-way ANOVA followed by Tukey's HSD post hoc test). **e** Tumor tissue TC levels in nude mice implanted with A549 cells after 15 days of 3 mg/kg cisplatin chemotherapy (n = 5 independent samples; one-way

ANOVA followed by Tukey's HSD post hoc test). **f** Cytotoxicity and IC_{50} of cisplatin were determined by the MTT assay in H1975, A549, and A549/DDP cells after 24 h incubation (*n* = 3 independent samples; one-way ANOVA followed by Tukey's HSD post hoc test). **g** Cytotoxicity and IC_{50} of cisplatin treatment on A549 cells were determined by MTT assay after 24 h incubation with cholesterol (*n* = 3 independent samples; one-way ANOVA followed by Tukey's HSD post hoc test). After Pifithrin-α (**h**), and STI571 (**i**) for 24 h, the cytotoxicity and IC_{50} of cisplatin treatment on A549 cells were measured by the MTT method, and then the enzymatic activity of caspas3/9 was detected (*n* = 3 independent samples; two-tailed unpaired t test for **h**(i, ii) and **i**(i, ii); one-way ANOVA followed by Tukey's HSD post hoc test for **h**(iv) and **i**(iv)). **j** Schematic of the mechanism by which cisplatin causes p53 mutations leading to malignancy after entering cells. Data are shown as the mean ± SD; n.s. = no significance. Source data are provided as a Source Data file. **j** created with BioRender.com.

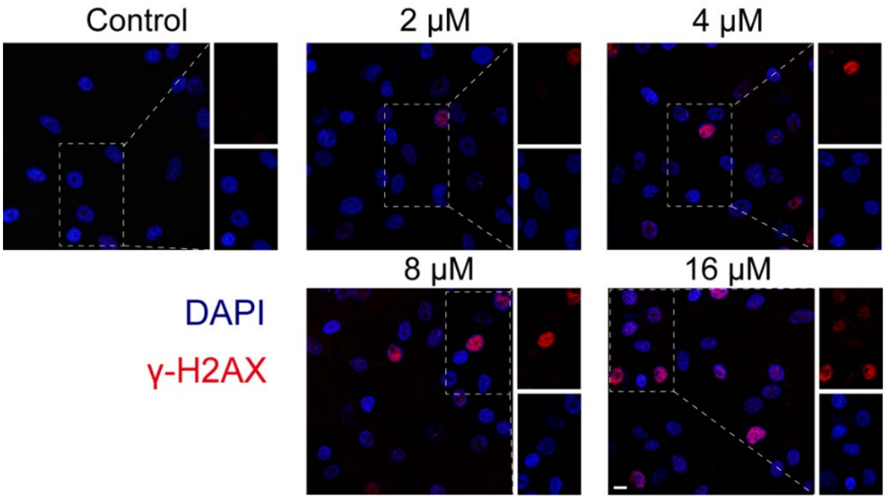

Supplementary Fig. 5 Expression of γ-H2AX after cisplatin treatment. Confocal images of IF staining against γ-H2AX in A549 cells treated with different concentrations of cisplatin for 12 h (*n* = 3 independent samples). Scale bars, 10 μm.

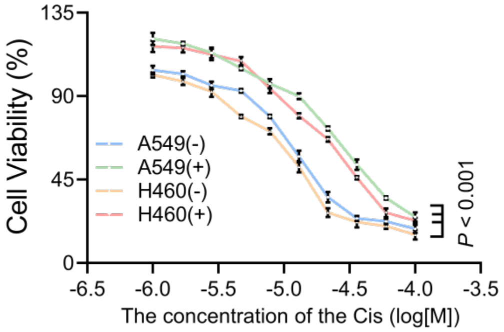

	IC_{50} (μM)	IC_{30} (μM)
A549(-)	15.62 ± 1.09	24.81 ± 1.34
A549(+)	38.92 ± 1.45	72.73 ± 2.03
H460(-)	12.94 ± 0.63	21.08 ± 1.88
H460(+)	33.34 ± 1.46	67.08 ± 2.69

Supplementary Fig. 6 Cytotoxicity of cisplatin in A549 cells and H460 cells with and without serum. With serum (-), without serum (+). (*n* = 3 independent samples; one-way ANOVA followed by Tukey's HSD post hoc test). Data are shown as the mean ± SD Source data are provided as a Source Data file.

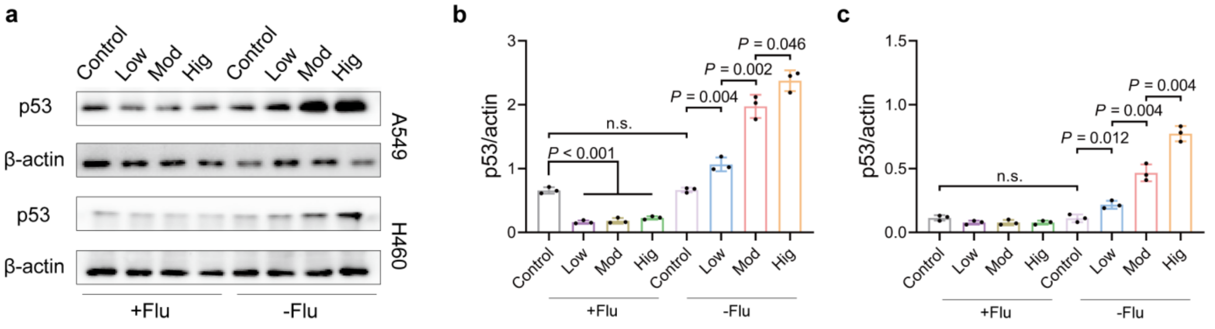

Supplementary Fig. 7 Western blotting analysis of p53 in cisplatin-induced cells after treatment with 4 μM fluvastatin for 12 h. a Western blotting analysis, and their fluorescence intensity were quantified (b-c). (*n* = 3 independent samples). Data are shown as the mean ± SD Statistical analysis was performed using two-tailed unpaired t test for b, c; n.s. = no significance. Source data are provided as a Source Data file.

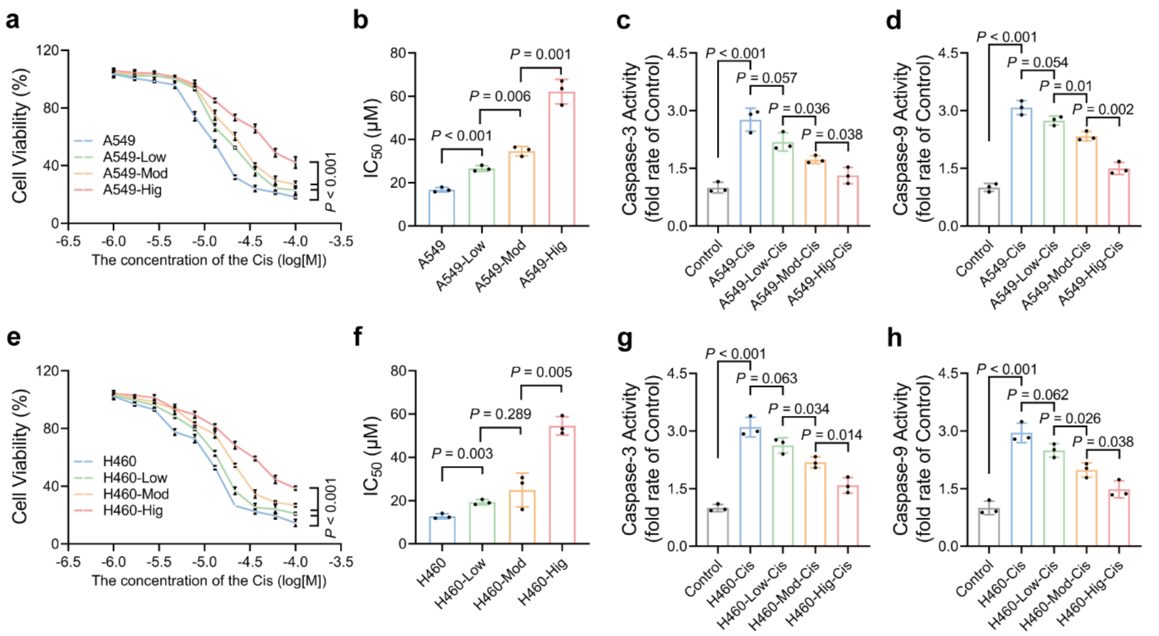

Supplementary Fig. 8 The cytotoxicity and Caspase enzymatic activity of A549 cells and H460 cells after cisplatin treatment. The cytotoxicity and IC_{50} of cisplatin treatment on A549 cells (**a**, **b***)* and H460 cells (**e**, **f***)* were measured by the MTT method, and then the enzymatic activity of Caspase-3 and Caspase-9 was detected (**c**, **d**, **g**, **h**) (n = 3 independent samples). Data were shown as the mean ± SD Statistical analysis was performed using two-tailed unpaired t test for c, d, g, and h; and one-way ANOVA followed by Tukey's HSD post hoc test for a, b, e, and f. Source data are provided as a Source Data file.

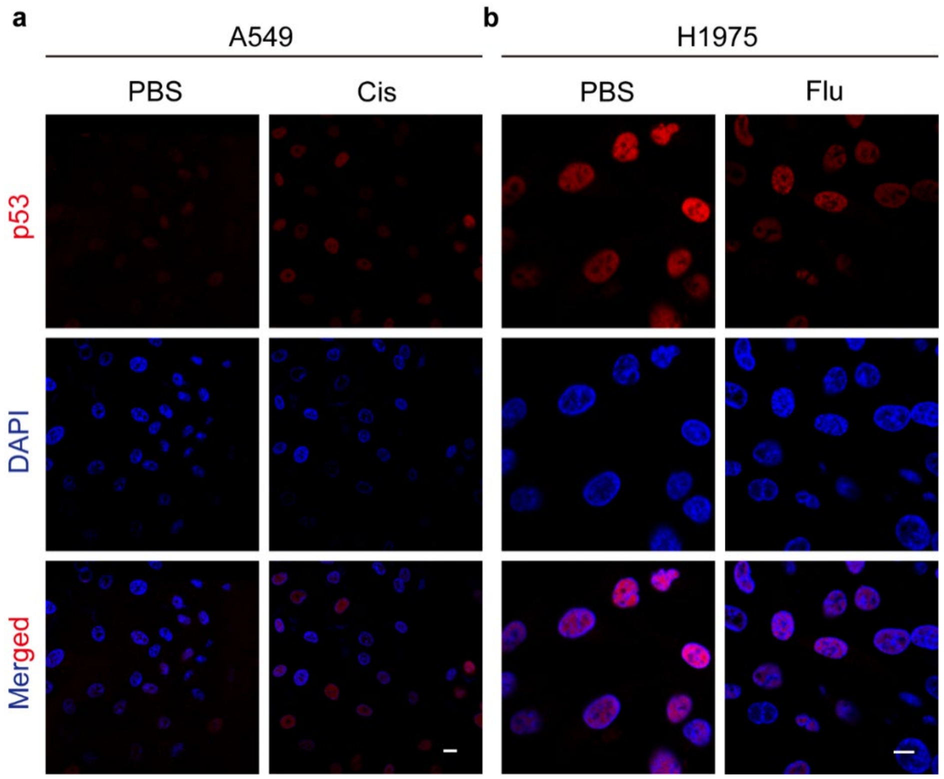

Supplementary Fig. 9 Expression of p53 after cisplatin or fluvastatin sodium treatment. **a** Confocal images of IF staining against p53 in A549 cells treated with 4 µM cisplatin for 12 h. Scale bars, 10 µm. **b** Confocal images of IF staining against p53 in H1975 cells treated with 8 µM fluvastatin sodium for 12 h. Scale bars, 10 µm. *n* = 3 independent samples.

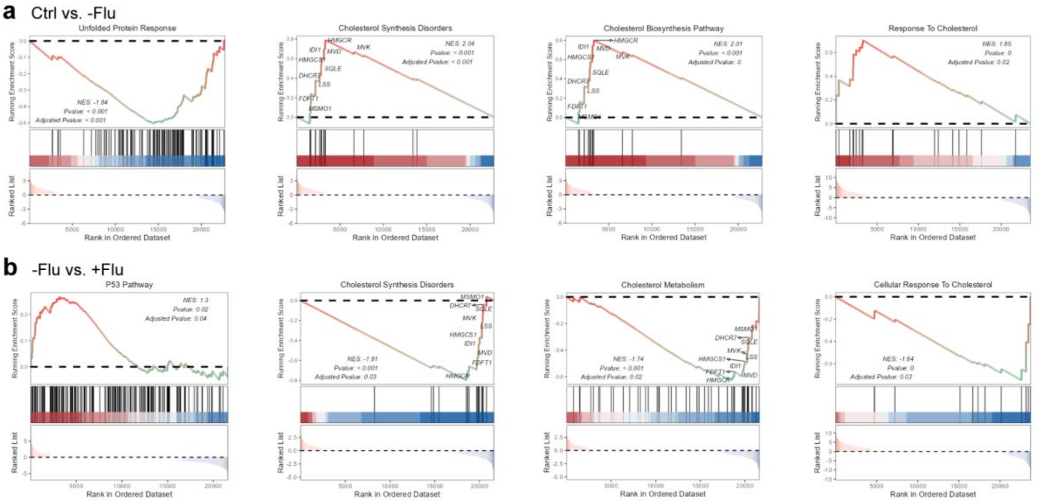

Supplementary Fig. 10 GSEA shows how Flu affects the gene signatures of cholesterol metabolism, the unfolded protein response, and the p53 pathway. **a** Analysis between control group and the group without Flu treatment. **b** Analysis between the groups with or without Flu treatment. The normalized enrichment scores (NES) and *p*-values are indicated in each plot.

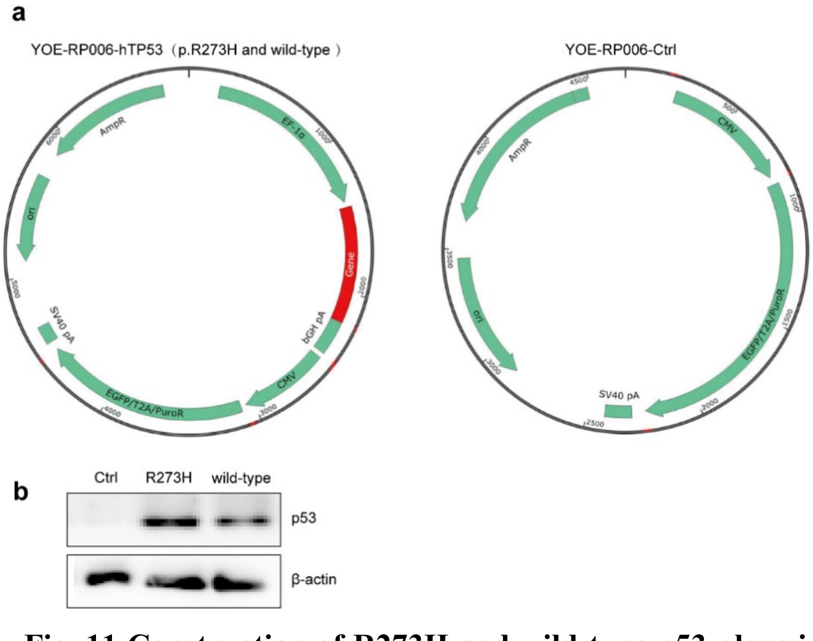

Supplementary Fig. 11 Construction of R273H and wild-type p53 plasmids. **a** Schematic diagram of the plasmid. **b** Immunoblotting in H1299 cells transfected with control, R273H,

and wild-type plasmids (n = 3 independent samples). Source data are provided as a Source Data file.

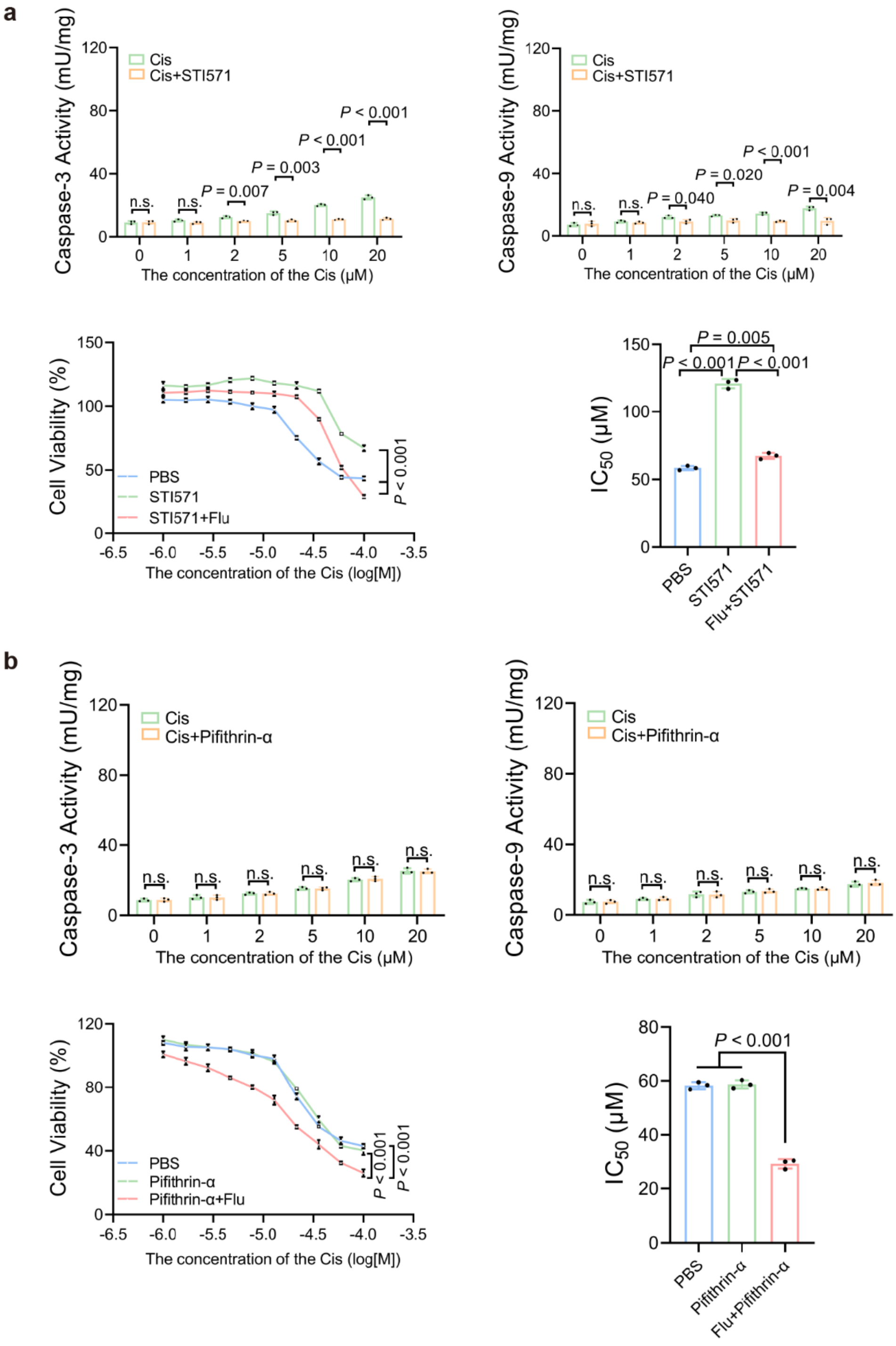

Supplementary Fig. 12 Cytotoxicity and Caspase enzymatic activity of H1975 cells after STI571 and Pifithrin-α treatment. After STI571 (**a**) and Pifithrin-α (**b**) treatment for 24 h, the cytotoxicity and IC_{50} of cisplatin treatment on H1975 cells were measured by MTT method, and then the enzymatic activity of Caspase-3 and Caspase-9 was detected (n = 3 independent samples). Data are shown as the mean ± SD Statistical analysis was performed using two-tailed unpaired t test for **a** (i, ii, iv) and **b** (i, ii, iv); one-way ANOVA followed by

Tukey's HSD post hoc test for **a** (iii) and **b** (iii); n.s. = no significance. Source data are provided as a Source Data file.

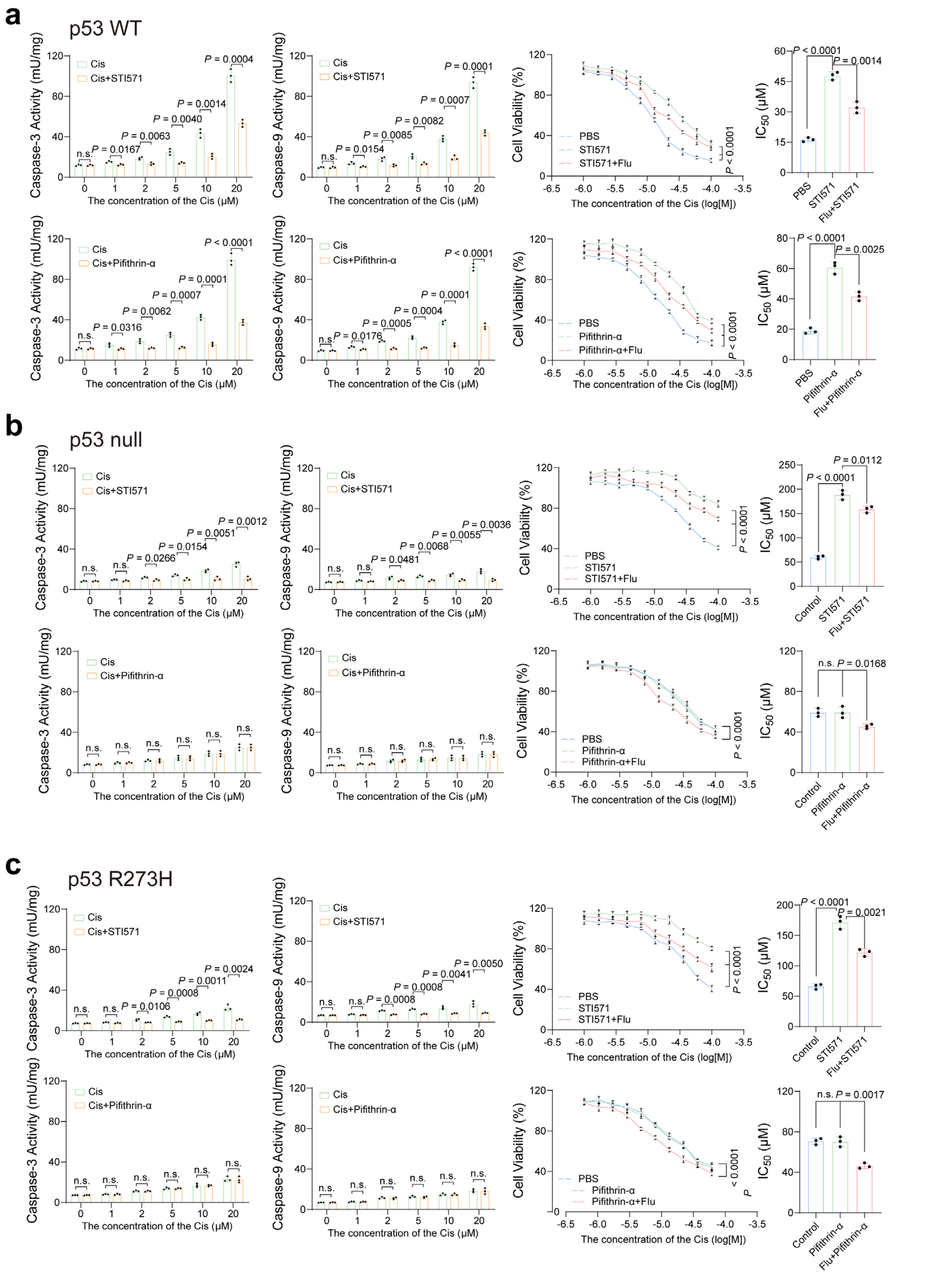

Supplementary Fig. 13 Cytotoxicity and Caspase enzymatic activity of H1299 cells after STI571 and Pifithrin-α treatment. After Pifithrin-α and STI571 treatment for 24 h, the cytotoxicity and IC_{50} of cisplatin treatment on in H1299 cells transfected with expression

constructs containing wtp53 (**a**), vector (**b**), and expression constructs containing the R273H variant (**c**) were measured by the MTT method, and then the enzymatic activity of caspas3/9 was detected (n = 3 independent samples; two-tailed unpaired t test for **a** (i, ii, iv), **b** (i, ii, iv) and **c** (i, ii, iv); one-way ANOVA followed by Tukey's HSD post hoc test for **a** (iii), **b** (iii) and **c** (iii)). Data are shown as the mean ± SD; n.s. = no significance. Source data are provided as a Source Data file.

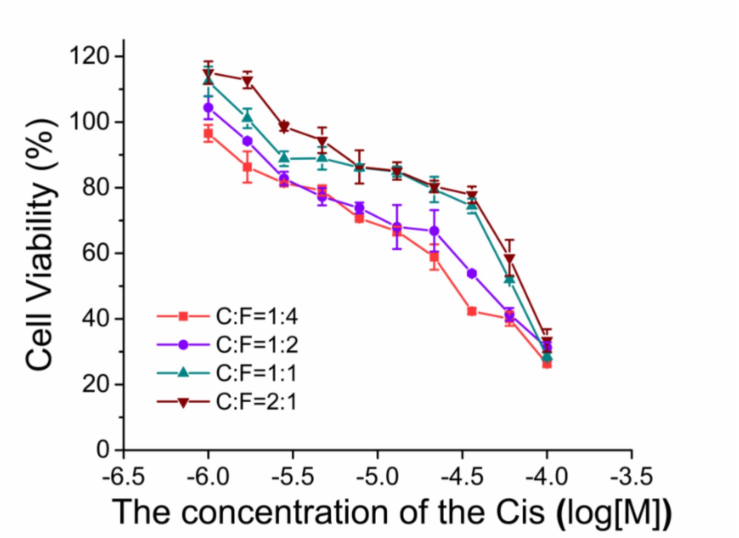

Supplementary Fig. 14 Cytotoxicity of different ratios of cisplatin and fluvastatin sodium. Cytotoxicity determined by the MTT assay in different ratios of physical mixture between cisplatin and fluvastatin sodium (n = 3 independent samples). Data are shown as the mean ± SD Source data are provided as a Source Data file.

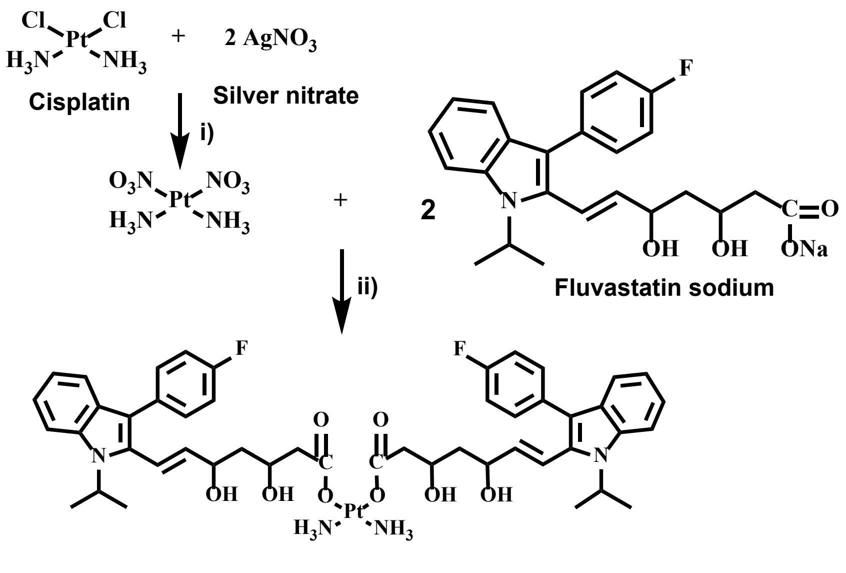

Supplementary Fig. 15 Synthetic route of Fluplatin. (i) Dechlorination reaction, (ii) Coordination reaction.

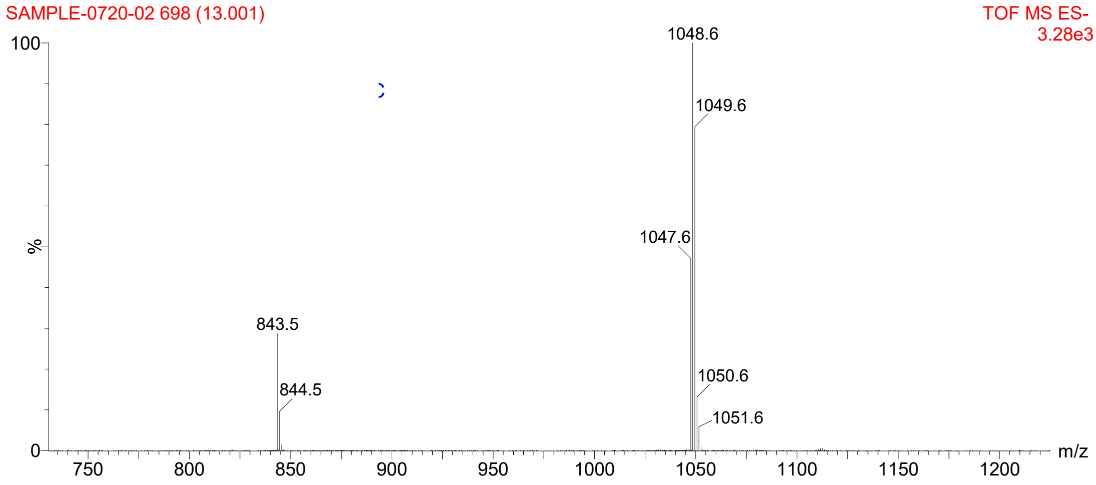

Supplementary Fig. 16 ESI-MS of Fluplatin. m/z=1048.6 $[M-H]^-$.

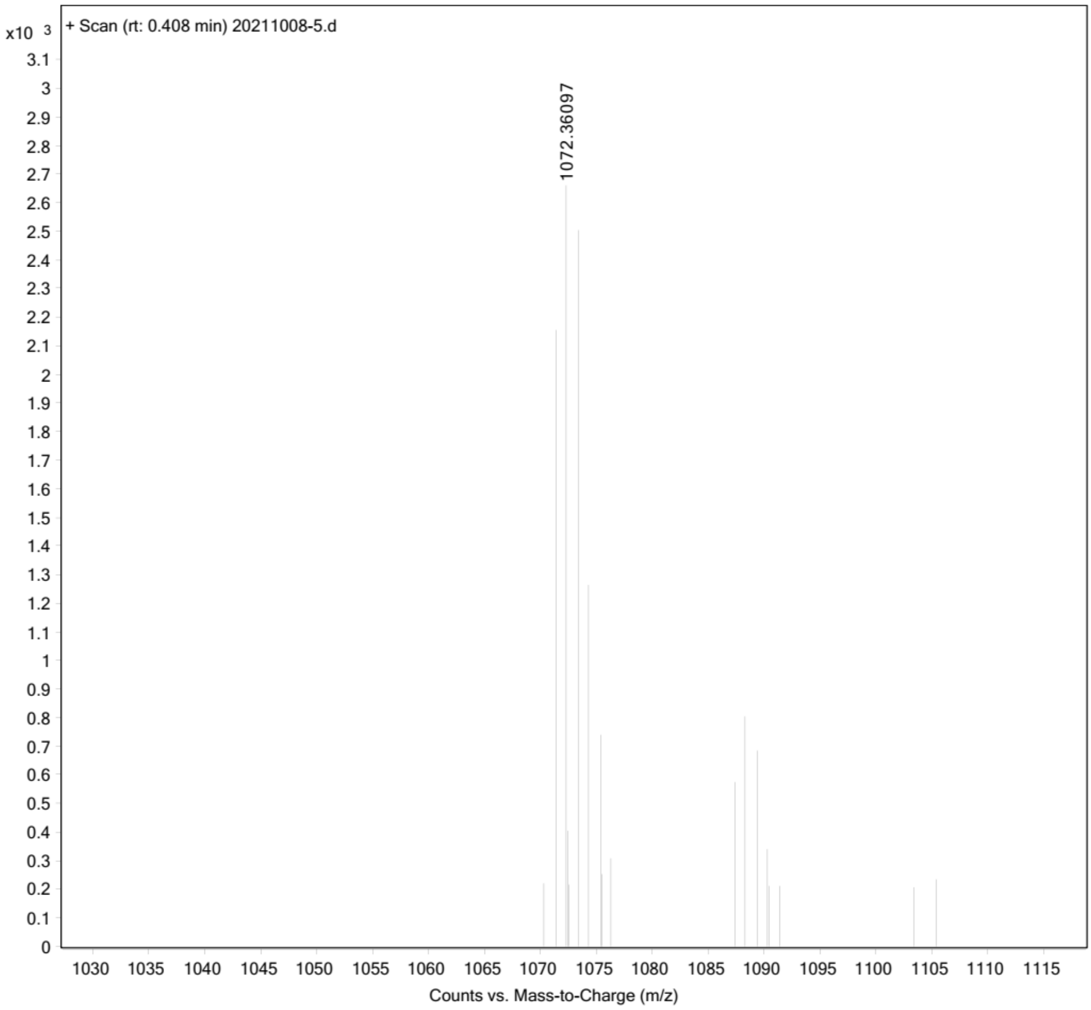

Supplementary Fig. 17 ESI-HRMS of Fluplatin. m/z=1072.36097 $[M+Na]^+$.

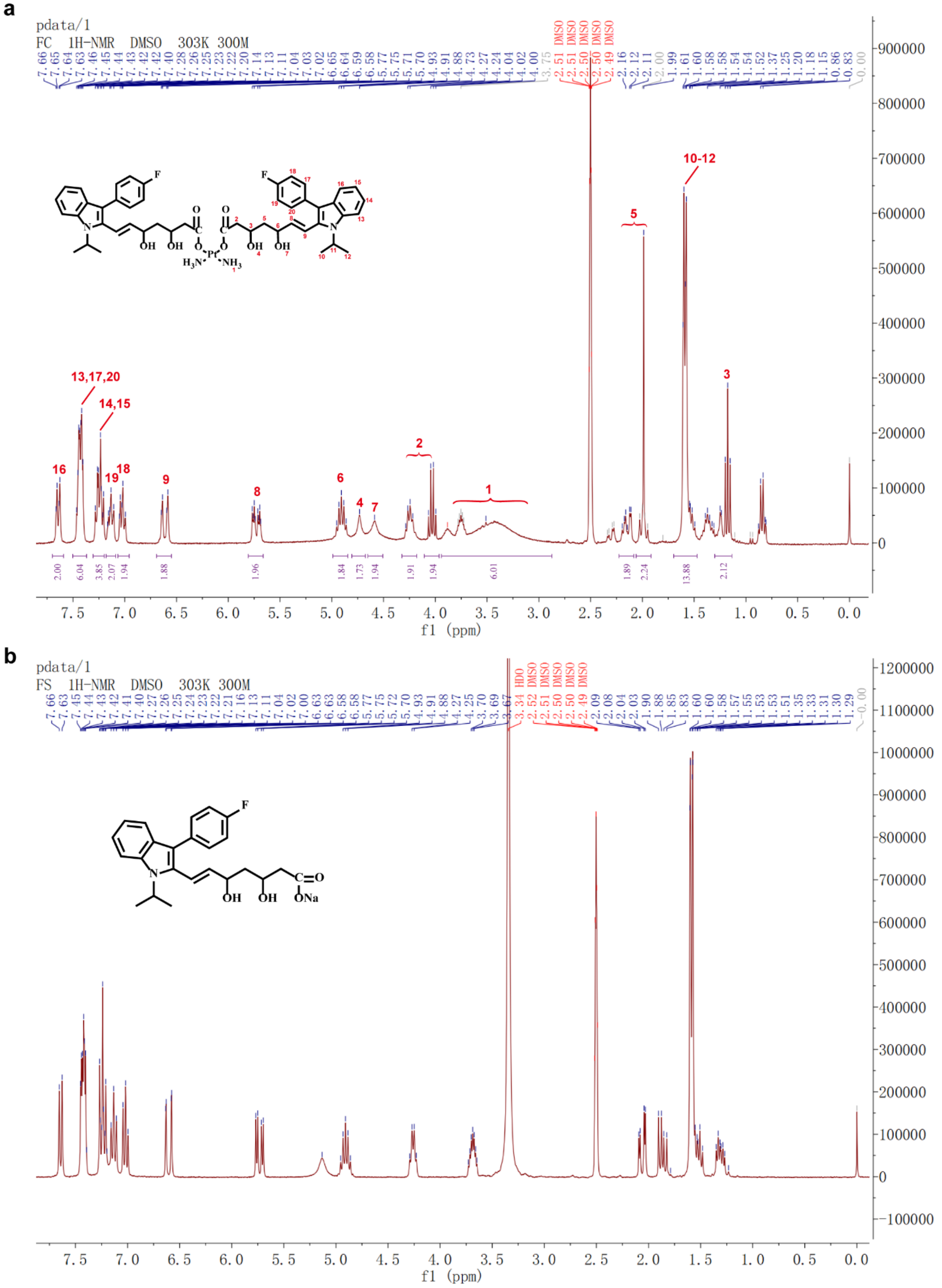

Supplementary Fig. 18 ^{1}H NMR spectra. Spectra of Fluplatin (**a**) and fluvastatin sodium (**b**) in DMSO-d6 (300 MHz). ^{1}H NMR (300 MHz, DMSO-d_6): δ ppm, 16-H: 7.64 (dd, J = 8.2, 2.8 Hz, 2H), 13,17,20-H: 7.43 (dt, J = 9.1, 3.0 Hz, 6H), 14,15-H: 7.24 (td, J = 8.8, 6.1 Hz, 4H), 19-H: 7.13 (dd, J = 9.1, 6.0 Hz, 2H), 18-H: 7.02 (dd, J = 8.8, 6.1 Hz, 2H), 9-H: 6.61 (dd, J =

15.8, 3.2 Hz, 2H), 8-H: 5.73 (ddd, J = 16.1, 5.8, 2.8 Hz, 2H), 6-H: 4.91 (p, J = 6.9 Hz, 2H), 4-H: 4.73 (s, 2H), 7-H: 4.59 (s, 2H), 2-H: 4.14 (m, 4H), 1-H: 4.82-2.71 (m, 6H), 5-H: 2.07 (m, 4H), 10,11,12-H: 1.59 (dd, J = 7.0, 2.5 Hz, 14H), 3-H: 1.29-1.24 (m, 2H).

a

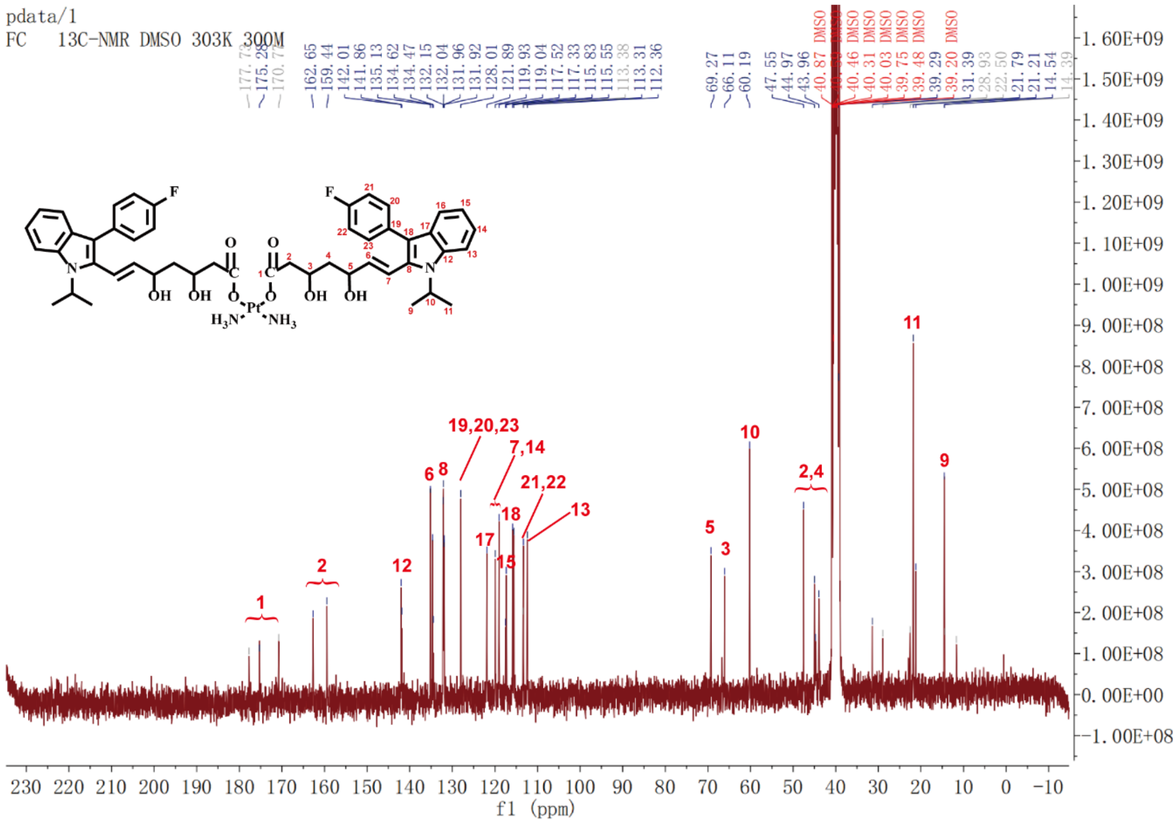

b

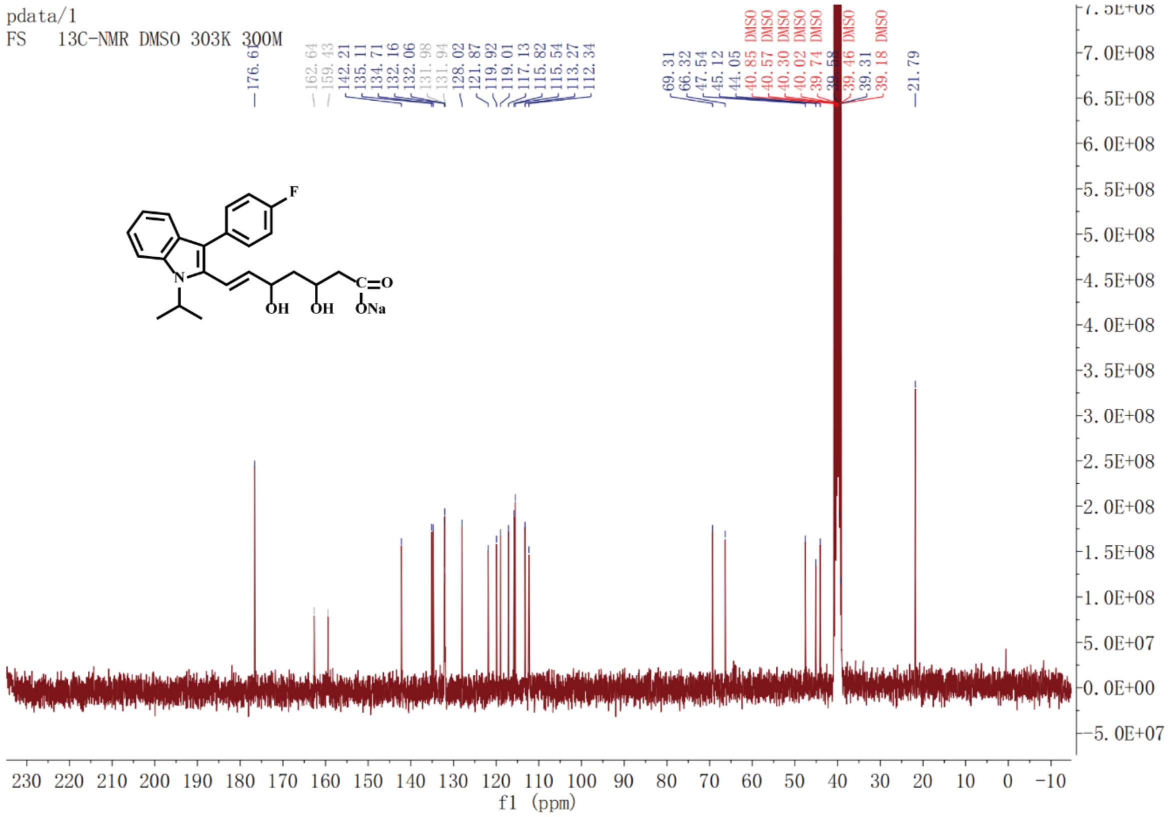

Supplementary Fig. 19 ^{13}C NMR spectra. Spectra of Fluplatin (**a**) and fluvastatin sodium (**b**) in DMSO-d6 (300 MHz).

a

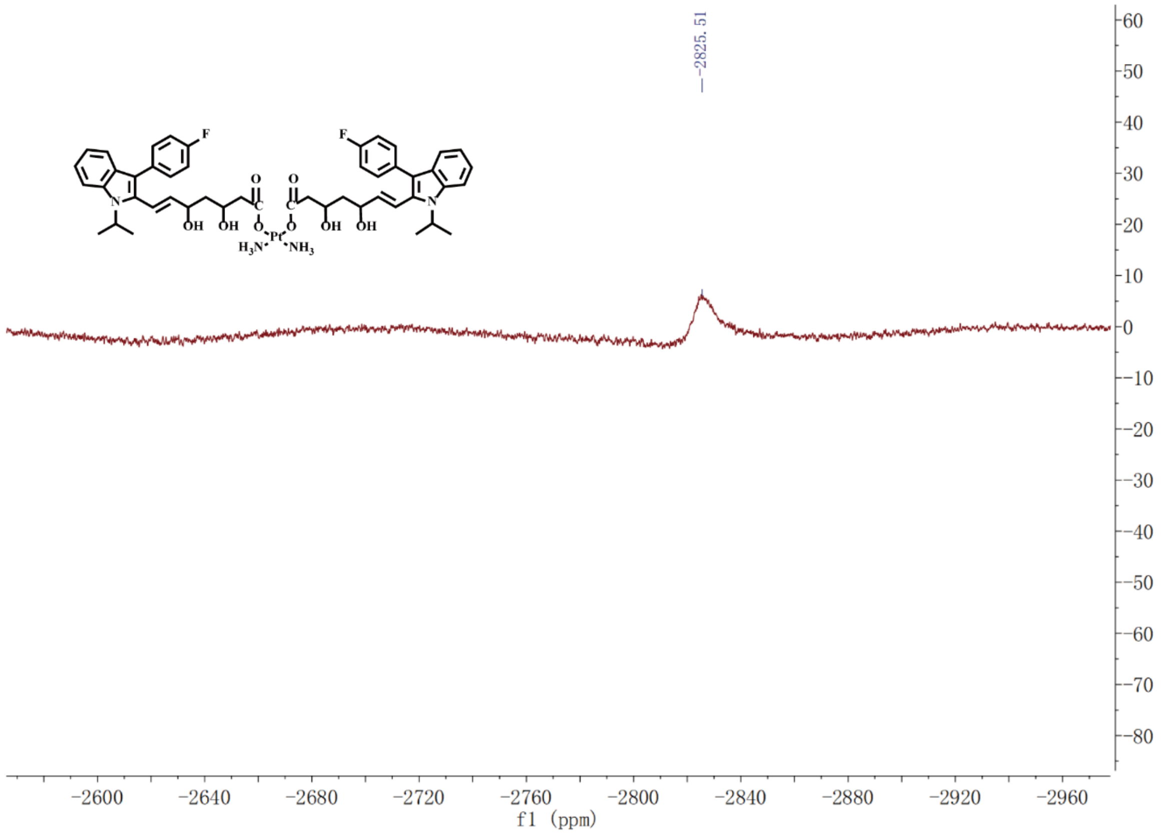

b

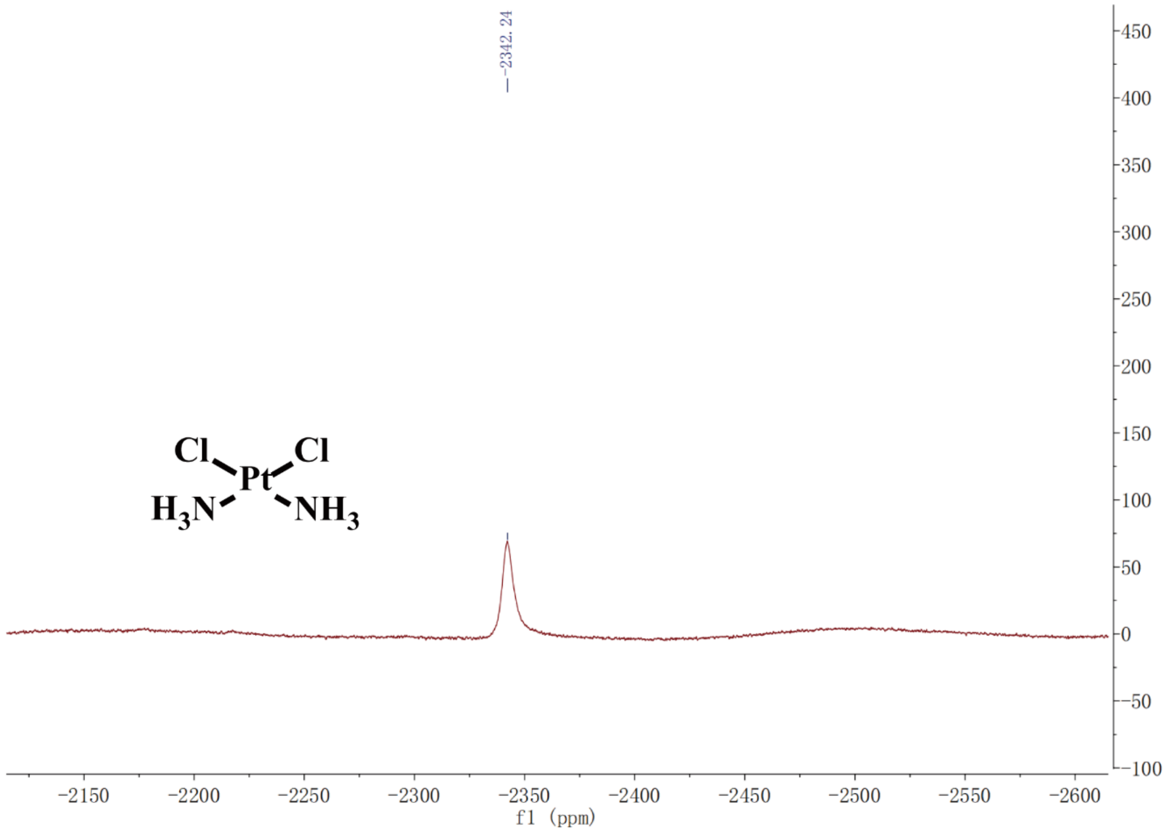

Supplementary Fig. 20 ^{195}Pt NMR spectra. Spectra of Fluplatin (**a**) and cisplatin (**b**) in DMSO-d_7.

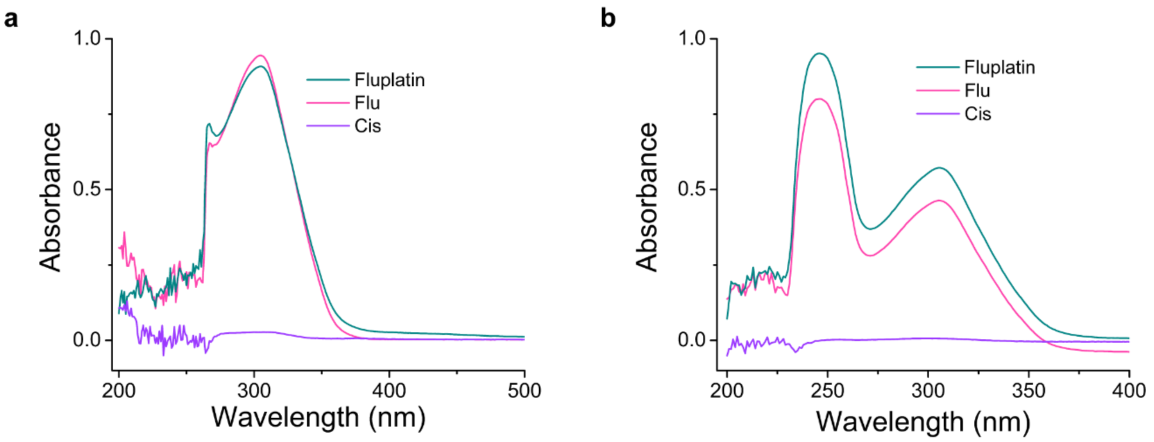

Supplementary Fig. 21 UV–vis spectra of Fluplatin, fluvastatin sodium and cisplatin. a The solvent was DMSO. **b** The solvent was methanol.

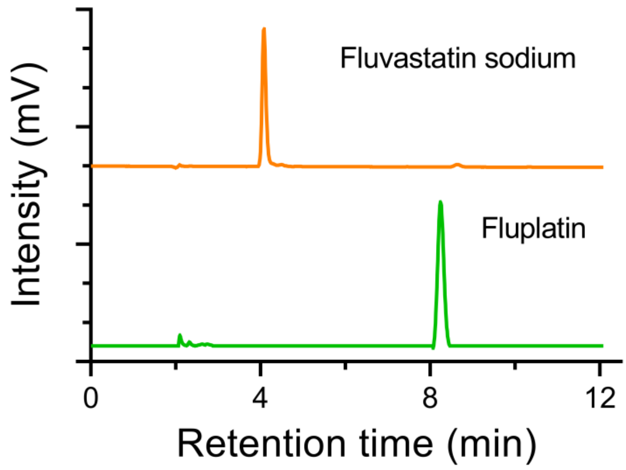

Supplementary Fig. 22 RP-HPLC (305 nm) chromatograms of Fluplatin and fluvastatin sodium.

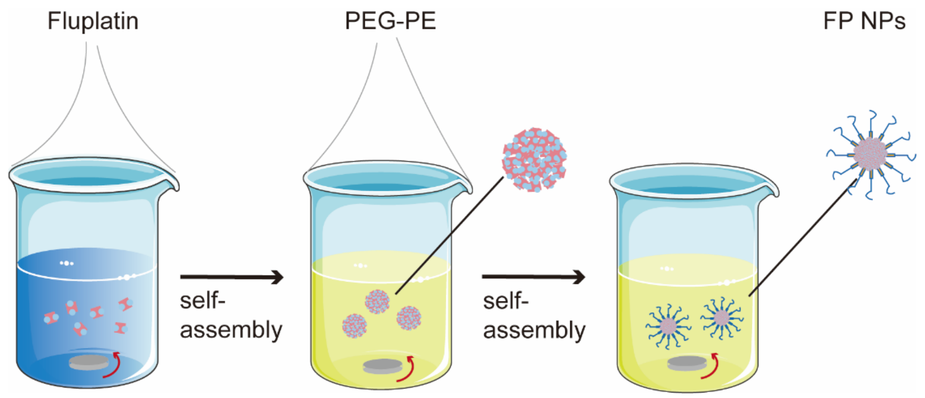

Supplementary Fig. 23 Preparation route of FP NPs.

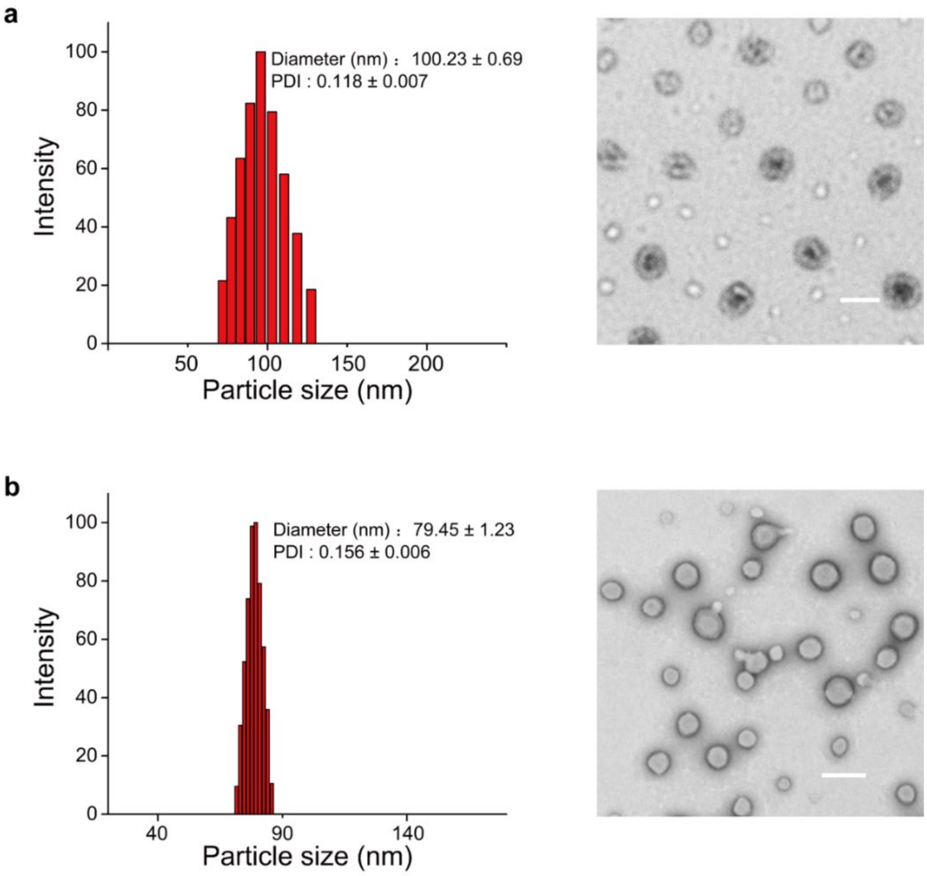

Supplementary Fig. 24 Particle size, PDI and TEM image of the different formulations. **a** The ratio of Fluplatin:PEG-PE was 10:1. **b** The ratio of Fluplatin:PEG-PE was 10:3. Scale bars, 200 nm. *n* = 3 independent samples.

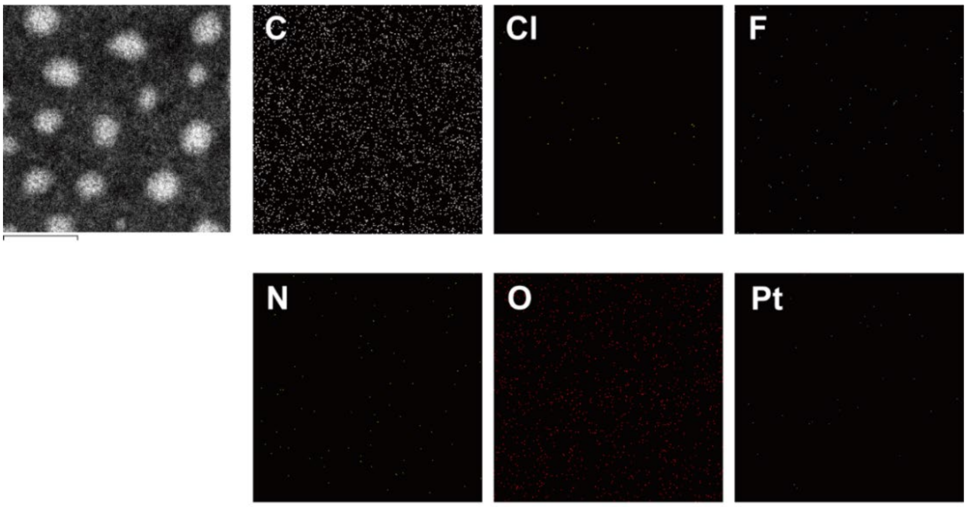

Supplementary Fig. 25 TEM image and EDS element mapping of FP NPs. Scale bars, 200 nm. n = 3 independent samples.

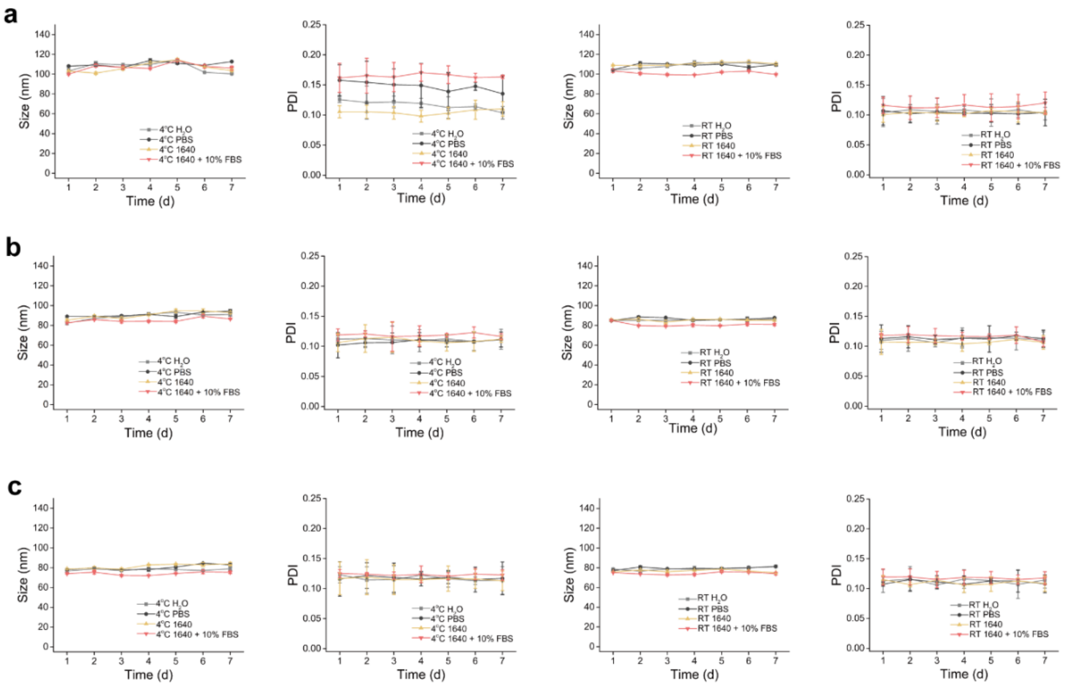

Supplementary Fig. 26 Storage stability of FP NPs. a The ratio of Fluplatin:PEG-PE was 10:1 (*n* = 3 independent samples). **b** The ratio of Fluplatin:PEG-PE was 5:1 (*n* = 3 independent samples). **c** The ratio of Fluplatin:PEG-PE was 10:3 (*n* = 3 independent samples). Data are shown as the mean ± SD Source data are provided as a Source Data file.

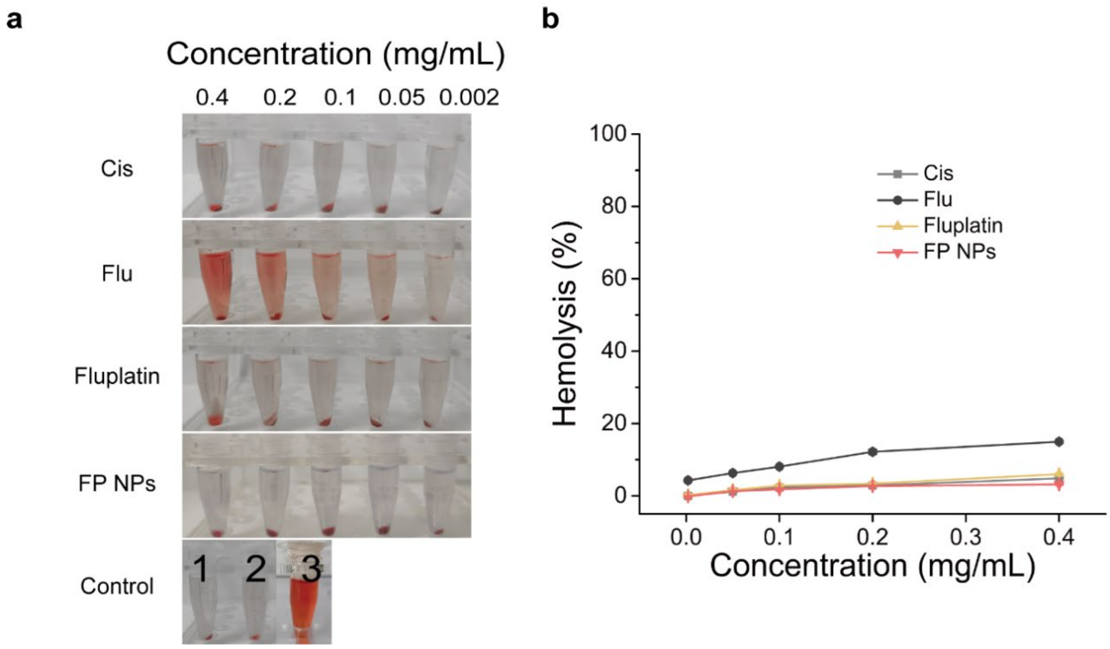

Supplementary Fig. 27 Hemolysis experiment of FP NPs. a The appearance of red blood cell suspensions after incubation with different concentrations of drugs, cisplatin dose as the standard. 1, 2, and 3 represent H_2O and red blood cell suspensions, glucose and red blood cell suspensions, Triton X-100 and red cell suspensions, respectively. **b** The hemolysis rate of

different concentrations of drugs was measured by UV-vis at 540 nm (n = 3 independent samples). Data are shown as the mean ± SD Source data are provided as a Source Data file.

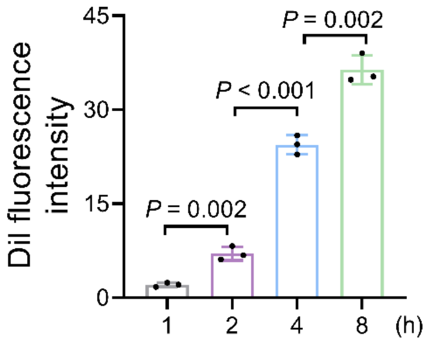

Supplementary Fig. 28 Quantification of Dil in H1975 cells after treatment with Dil@FP NPs. Data are shown as the mean ± SD Statistical analysis was performed using one-way ANOVA followed by Tukey’s HSD post hoc test; n.s. = no significance. Source data are provided as a Source Data file.

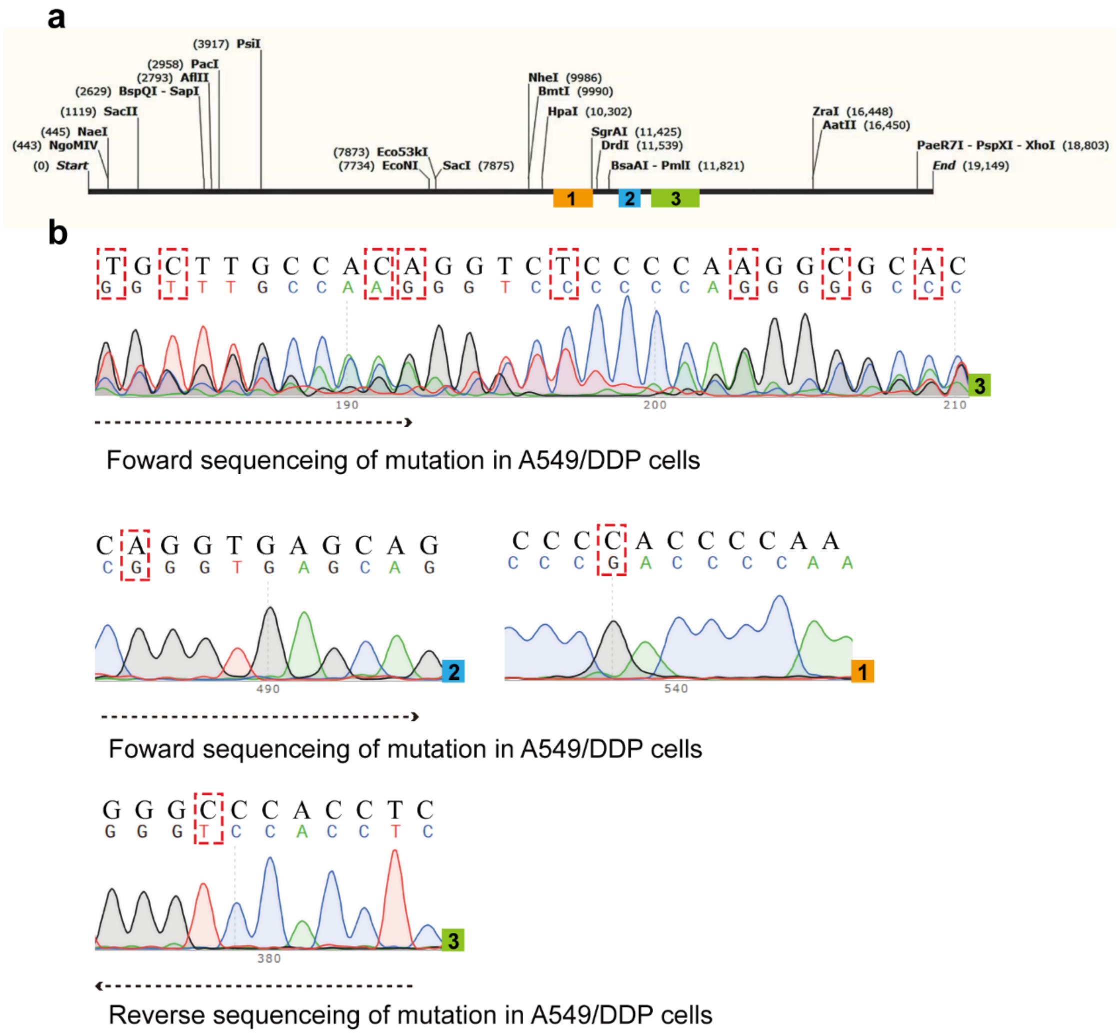

Supplementary Fig. 29 Gene sequencing of *TP53* mutation. a *TP53* gene sequence. **b** Sequencing in A549/DDP cells. Source data are provided as a Source Data file.

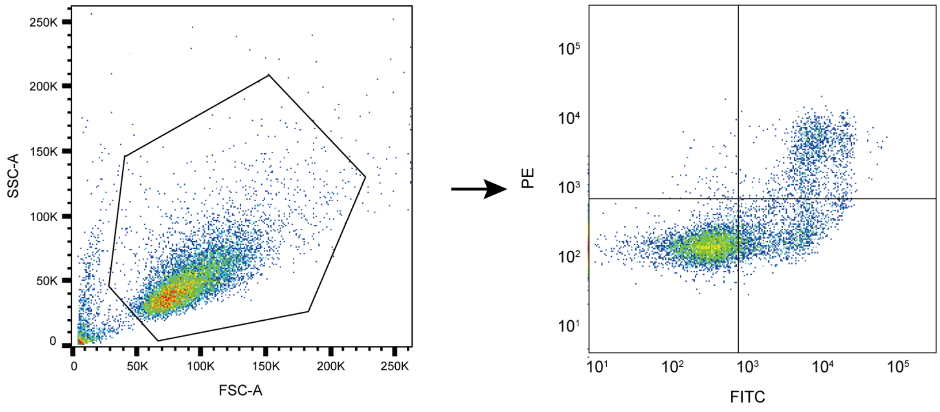

Supplementary Fig. 30 Gating strategy of The Apoptosis rate of H1975 cells.

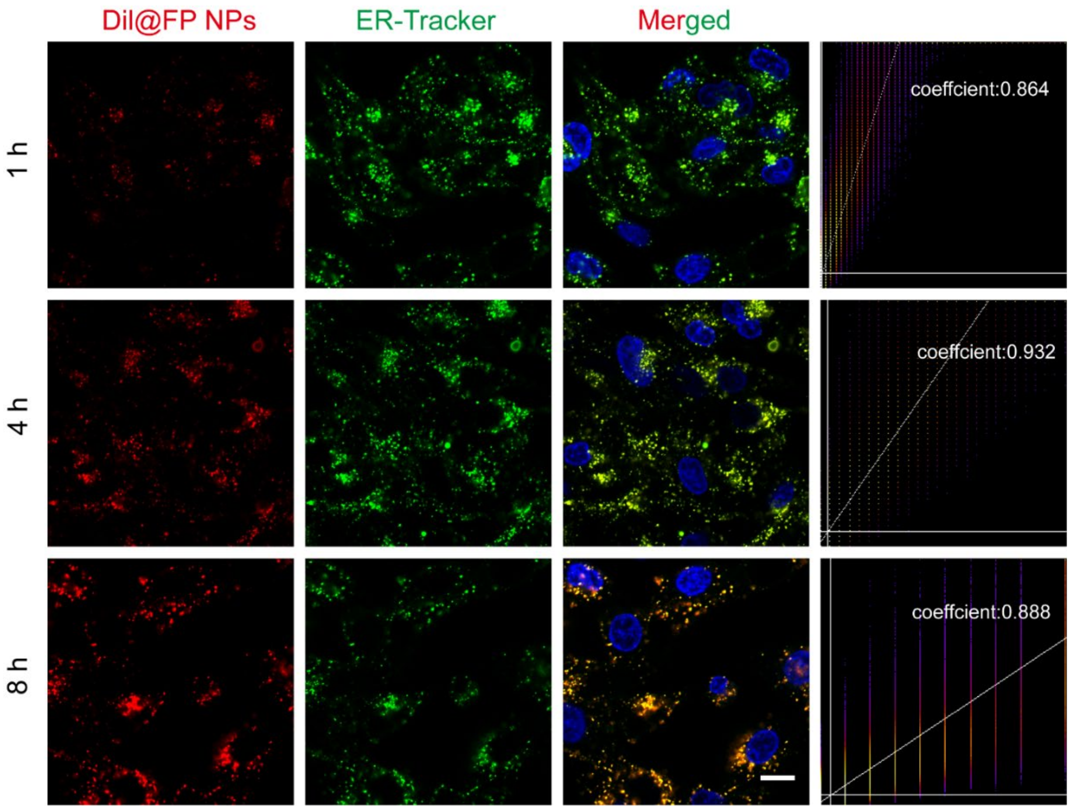

Supplementary Fig. 31 Colocalization results of Dil@FP NPs and ER in A549 cells. Confocal images of Dil and ER-Tracker in A549 cells treated with 2 μM Dil@FP NPs for 1 h, 4 h, and 8 h. Their colocalization determined by Pearson's correlation coefficient was quantified. $n = 3$ independent samples. Scale bars, 10 μm.

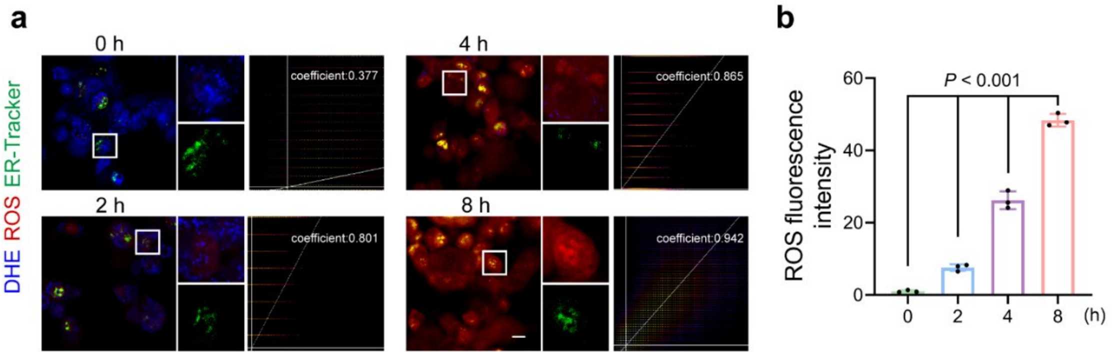

Supplementary Fig. 32 Colocalization results of ROS and ER in H1975 cells. a Confocal images of ROS and ER-Tracker in H1975 cells treated with 4 μM FP NPs for 1 h, 4 h, and 8 h. Their colocalization determined by Pearson's correlation coefficient was quantified. **b** ROS fluorescence intensity was quantified. Scale bars, 10 μm. *n* = 3 independent samples. Data are shown as the mean ± SD Statistical analysis was performed using one-way ANOVA followed by Tukey's HSD post hoc test; n.s. = no significance. Source data are provided as a Source Data file.

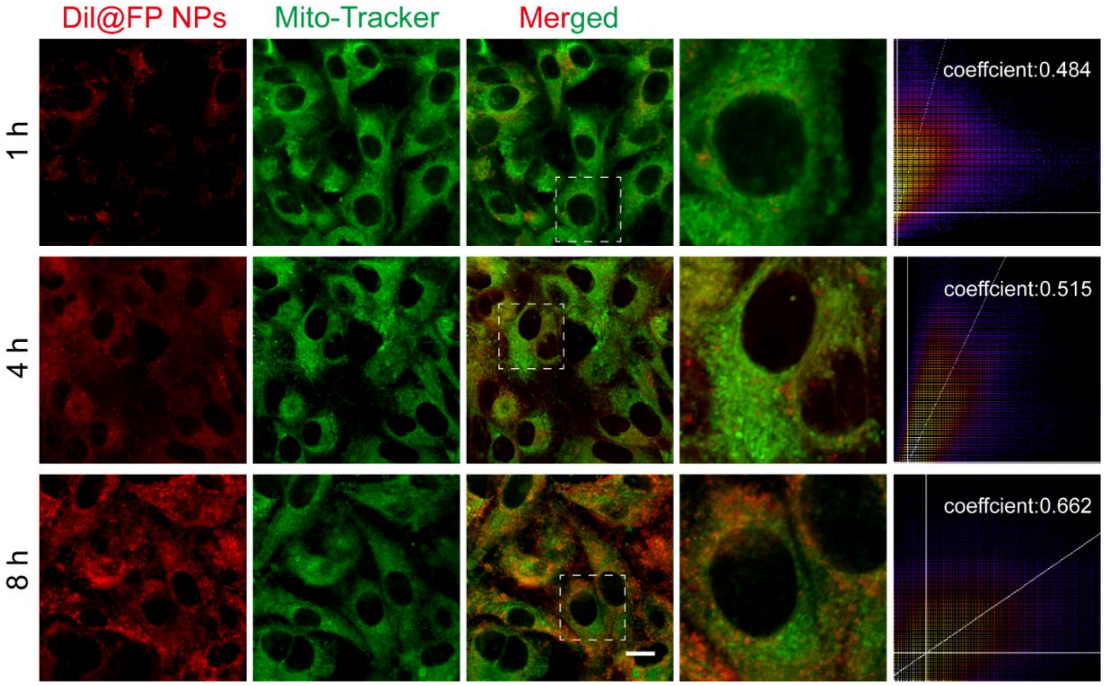

Supplementary Fig. 33 Colocalization results of Dil@FP NPs and Mito in A549 cells. Confocal images of Dil and MitoTracker in H1975 cells treated with 2 μM Dil@FP NPs for 1 h, 4 h, and 8 h, and their colocalization determined by Pearson's correlation coefficient was quantified. *n* = 3 independent samples. Scale bars, 10 μm.

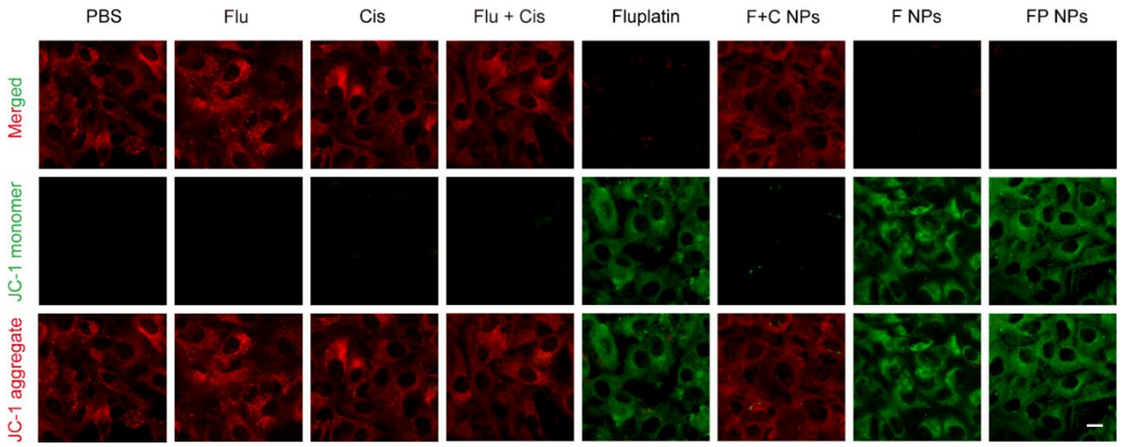

Supplementary Fig. 34 Confocal images of JC-1 in A549 cells treated with FP NPs. A549 cells treated with 4 μM FP NPs for 6 h. n = 3 independent samples. Scale bars, 10 μm.

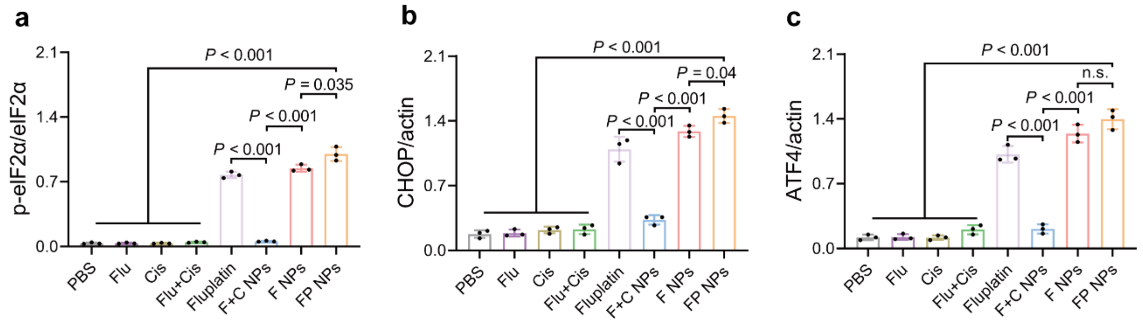

Supplementary Fig. 35 Quantification of Western blotting analysis. Analysis of p-elF2α (**a**), CHOP (**b**), and ATF4 (**c**) in H1975 cells after treatment with different formulations. Data are shown as the mean ± SD Statistical analysis was performed using one-way ANOVA followed by Tukey's HSD post hoc test; n.s. = no significance. Source data are provided as a Source Data file.

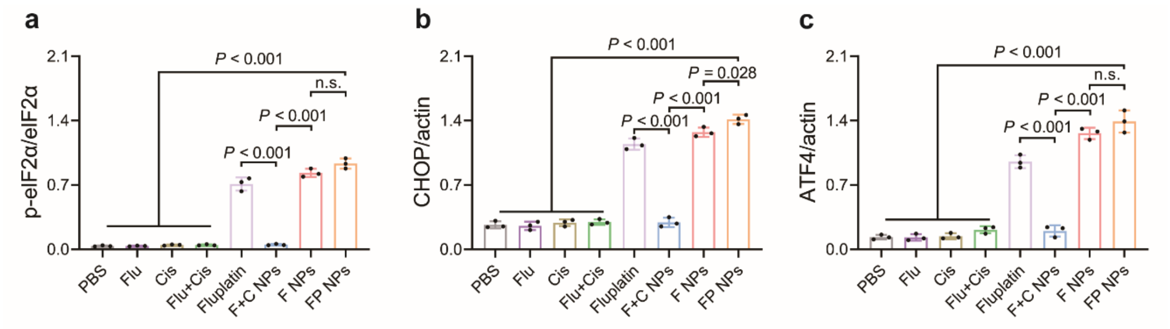

Supplementary Fig. 36 Quantification of Western blotting analysis. Analysis of p-elF2α (**a**), CHOP (**b**), and ATF4 (**c**) in A549 cells after treatment with different formulations. Data are shown as the mean ± SD Statistical analysis was performed using one-way ANOVA followed by Tukey's HSD post hoc test; n.s. = no significance. Source data are provided as a Source Data file.

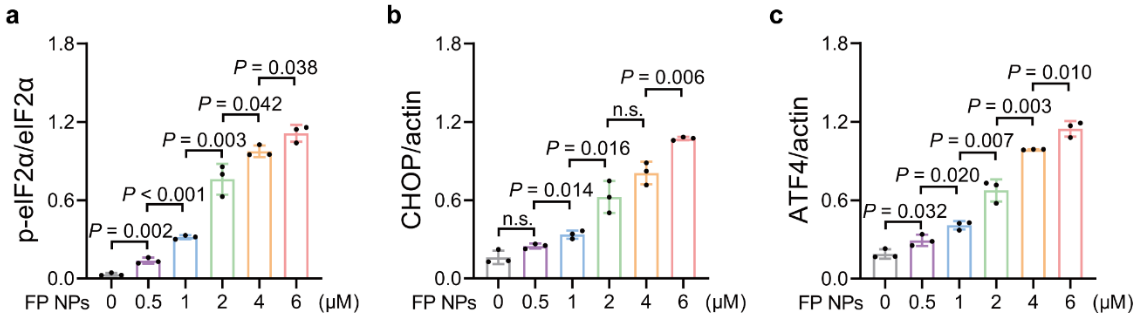

Supplementary Fig. 37 Quantitative of Western blotting analysis. Analysis of p-elF2α (**a**), CHOP (**b**), and ATF4 (**c**) in H1975 cells after treatment with different concentrations of FP NPs. Data are shown as the mean ± SD Statistical analysis was performed using one-way ANOVA followed by Tukey's HSD post hoc test; n.s. = no significance. Source data are provided as a Source Data file.

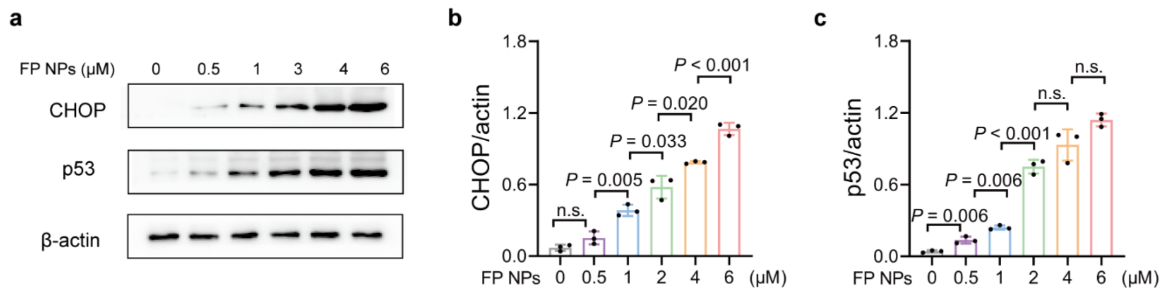

Supplementary Fig. 38 Western blotting analysis. Analysis of CHOP and p53 in A549 cells after treatment with different concentrations of FP NPs (**a**). Their grayscale values were quantified (**b**-**c**). *n* = 3 independent samples. Data are shown as the mean ± SD Statistical analysis was performed using one-way ANOVA followed by Tukey's HSD post hoc test; n.s. = no significance. Source data are provided as a Source Data file.

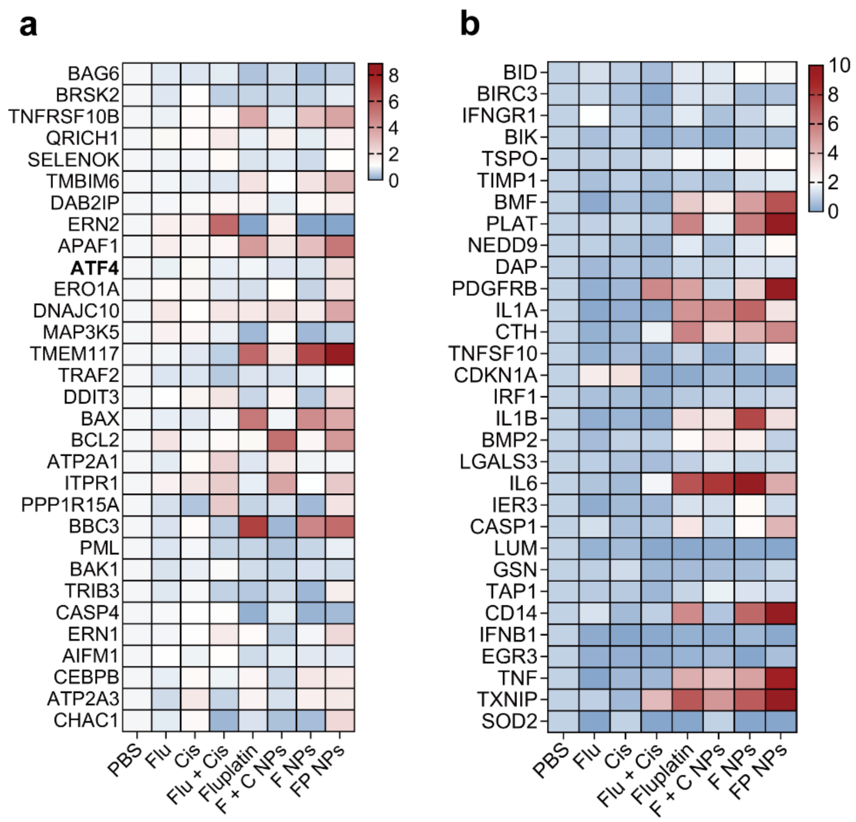

Supplementary Fig. 39 Heatmap analysis of ERS pathway genes (**a**) and apoptosis pathway genes (**b**) from RNA-seq data using each group of untreated A549 cells after cisplatin induction. The color scale indicates the fold change of genes.

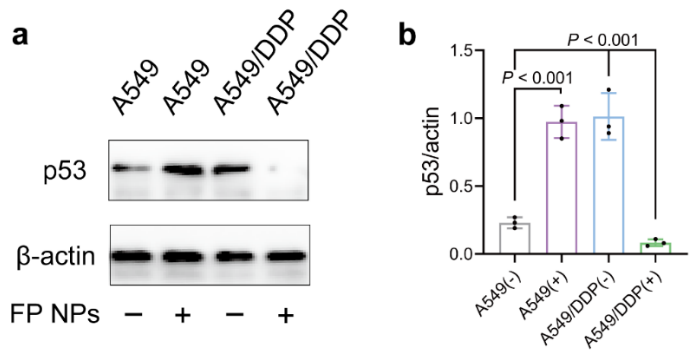

Supplementary Fig. 40 Western blotting analysis of p53 in A549 cells and A549/DDP cells after treatment with 4 μM FP NPs for 12 h. a Western blotting analysis, and their fluorescence intensity was quantified (b). (n = 3 independent samples). n = 3 independent samples. Data were shown as the mean ± SD Statistical analysis was performed using two-tailed unpaired t test for b. Source data are provided as a Source Data file.

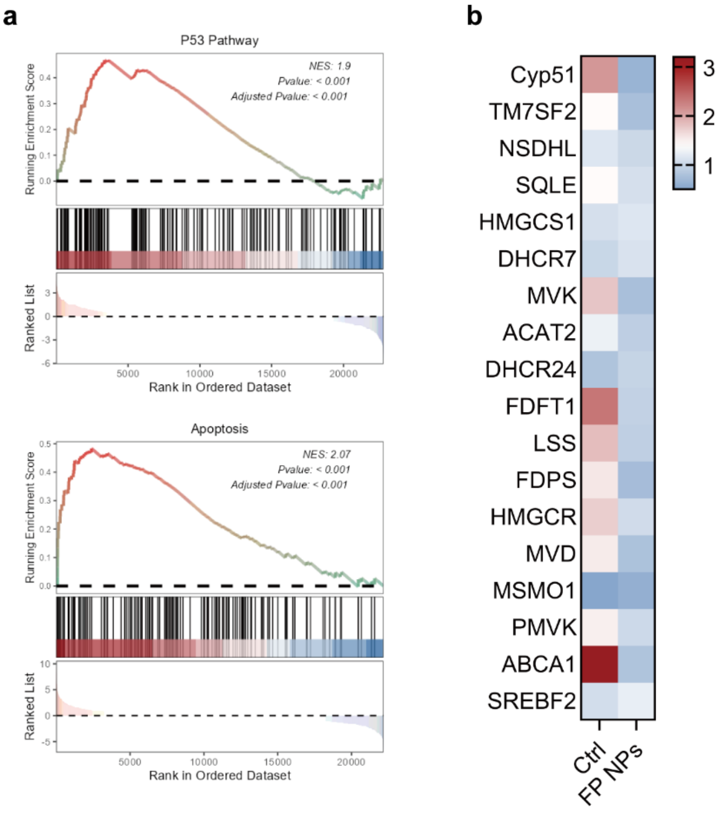

Supplementary Fig. 41 a GSEA shows how Flu affects the gene signatures of the p53 pathway and apoptosis. The normalized enrichment scores (NES) and *p*-values are indicated in each plot. **b** Heatmap analysis of 17 mevalonate pathway genes and the SREBP2 gene from RNA-seq data using fluvastatin sodium treated and untreated A549 cells after cisplatin induction. The color scale indicates the fold change of genes expression.

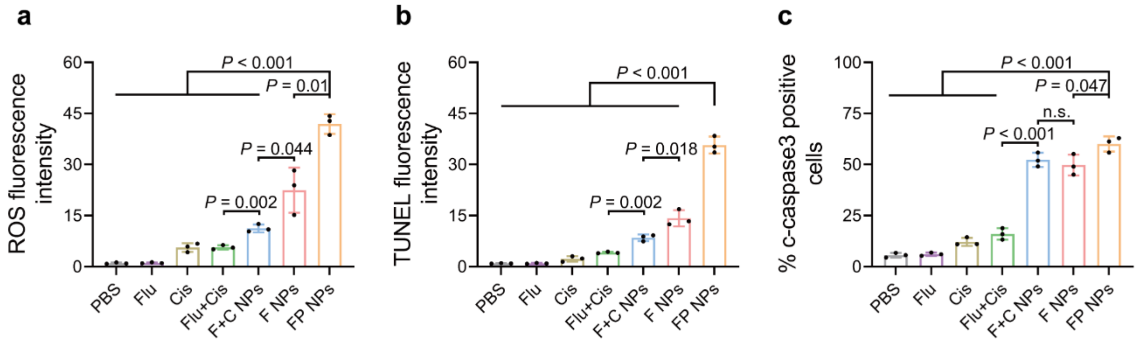

Supplementary Fig. 42 a Quantification of ROS fluorescence intensity after treatment with different formulations. **b** Quantification of TUNEL fluorescence intensity after treatment with different formulations. **c** Quantitative of c-caspase3 positive cells after treatment with different formulations. Data are shown as the mean ± SD Source data are provided as a Source Data file.

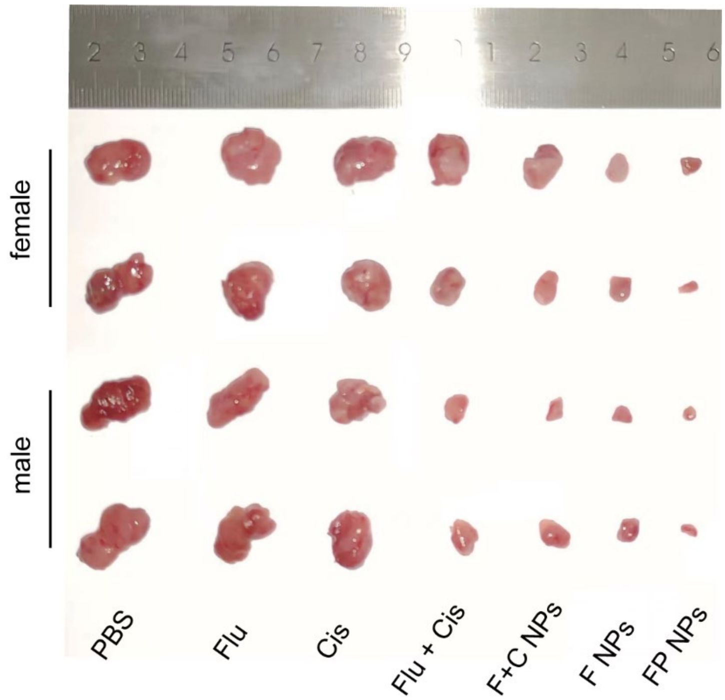

Supplementary Fig. 43 Antitumor efficacy of intravenously injected FP NPs in an H1975 tumor-bearing mouse model of different genders.

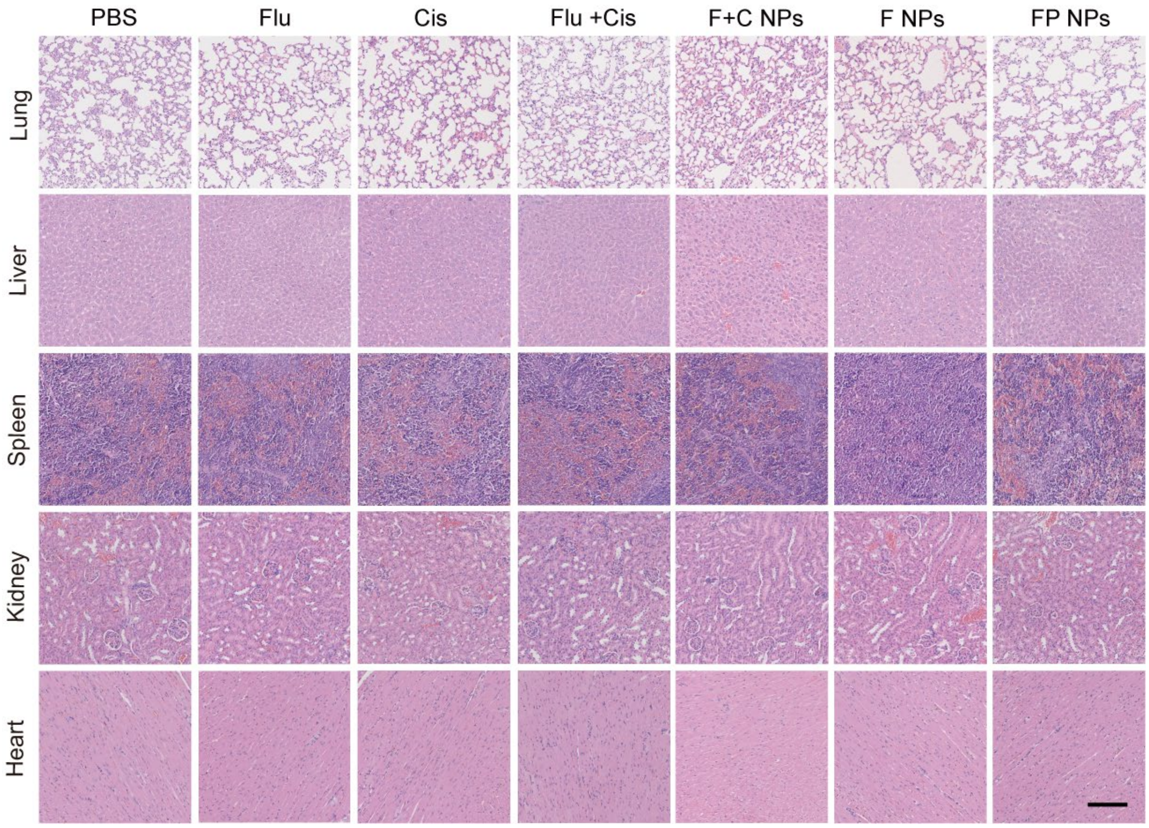

Supplementary Fig. 44 Histological analyses of the heart, liver, spleen, lung and kidneys were performed to evaluate the toxicity of different formulations. *n* = 3 independent samples. Scale bars, 200 μm.

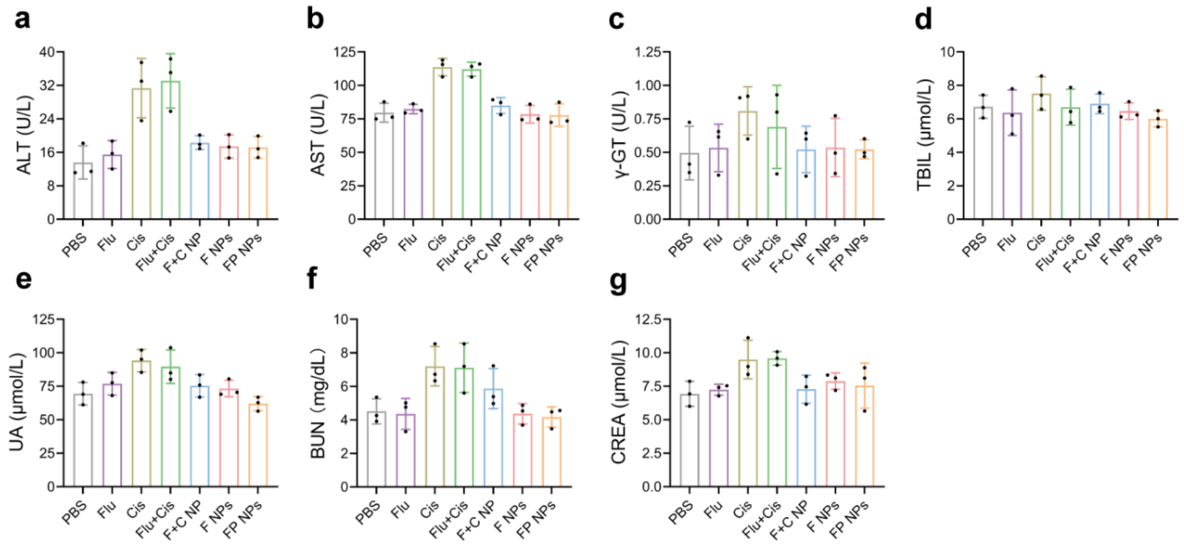

Supplementary Fig. 45 ALT (**a**), AST (**b**), γ-GT (**c**), TBIL (**d**), UA (**e**), BUN (**f**), and CREA (**g**) levels in serum. *n* = 3 independent samples. Data are shown as the mean ± SD Source data are provided as a Source Data file.

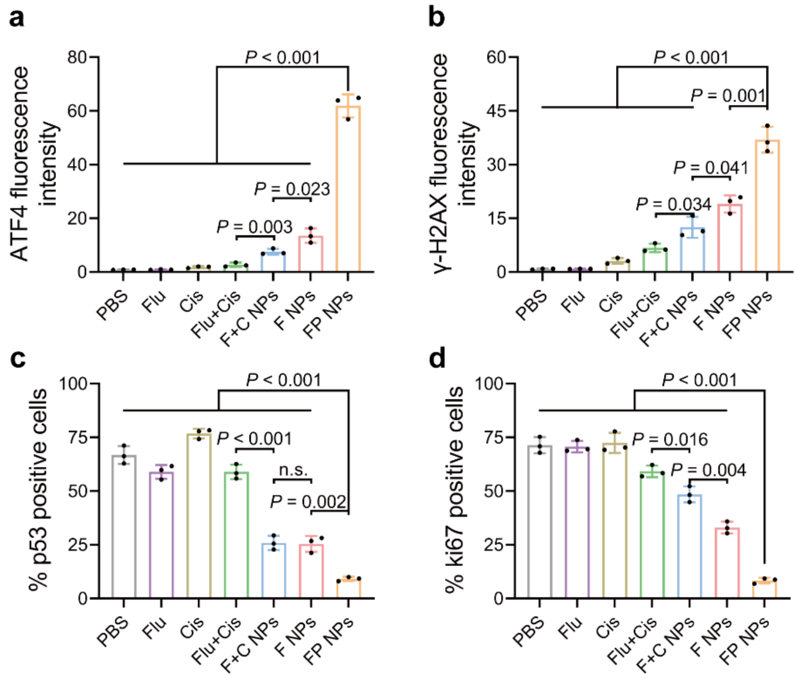

Supplementary Fig. 46 a Quantification of ATF4 fluorescence intensity after treatment with different formulations. **b** Quantification of γ-H2AX fluorescence intensity after treatment with different formulations. **c** Quantification of p53 positive cells after treatment with different formulations. **d** Quantification of ki67 positive cells after treatment with different formulations. Data are shown as the mean ± SD Source data are provided as a Source Data file.

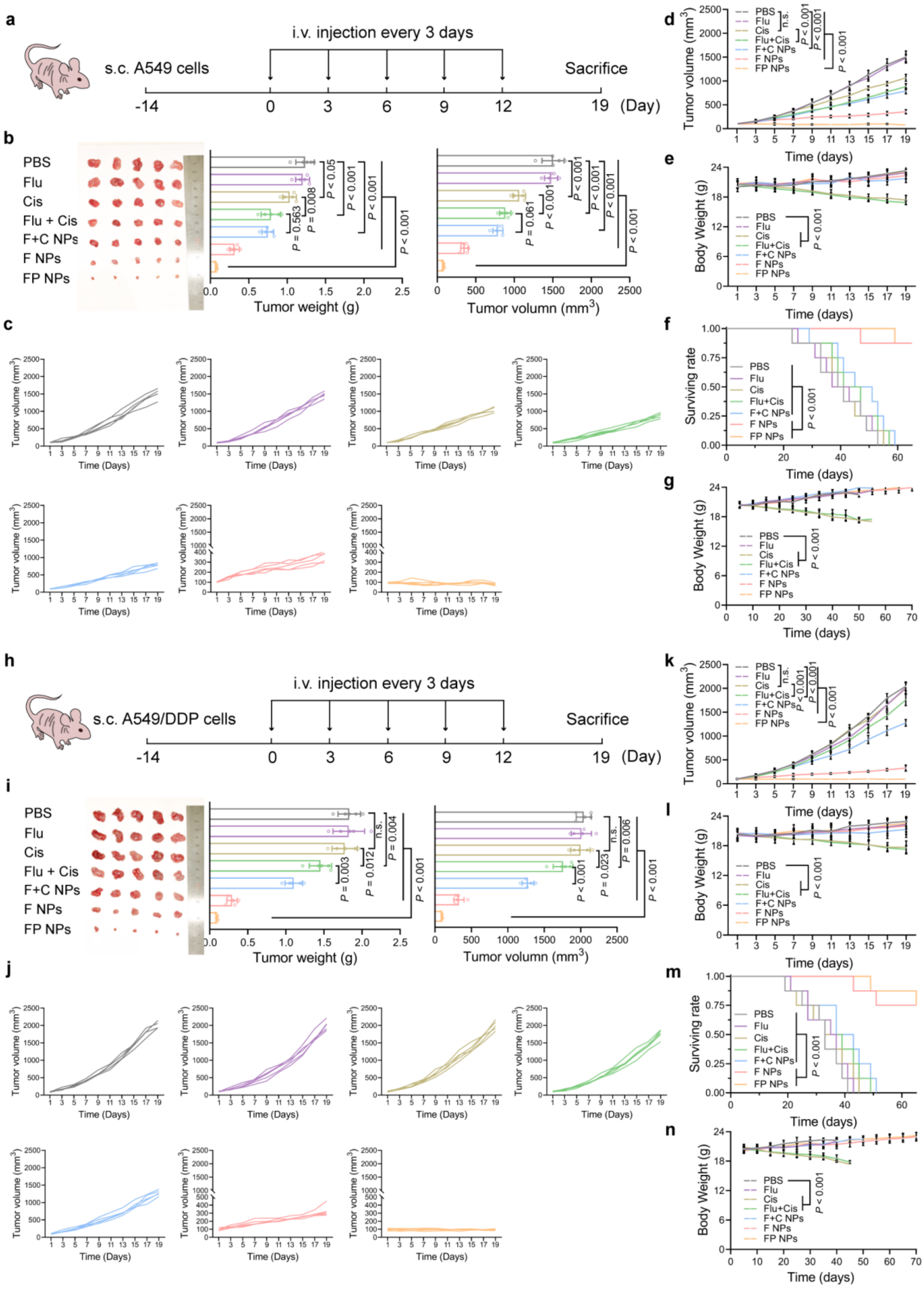

Supplementary Fig. 47 Antitumor efficacy of intravenously injected FP NPs in A549 and A549/DDP tumor-bearing mouse models. a, **h** Schematic illustration of the experimental design. **b**, **i** Images of tumor and tumor weight/volume on day 19 of each group (n = 5 mice per group; one-way ANOVA followed by Tukey's HSD post hoc test). **c**, **j** Tumor volume changes for each mouse in each group over 21 days of treatment (n = 5 mice per group). **d, k** The tumor volumes of mice during treatments with different formulations (n = 5 mice per group; two-way ANOVA followed by Tukey's multiple comparisons post test). **e, l** The body

weight of mice during treatments with different formulations (n = 5 mice per group; two-way ANOVA followed by Tukey's multiple comparisons post test). **f**, **m** Kaplan-Meier survival curve of mice treated with different formulations over 70 days (n = 8 mice per group; Log-rank Mantel–Cox test). **g**, **n** The body weight of mice during treatments with different formulations over 70 days (n = 8 mice per group;two-way ANOVA followed by Tukey's multiple comparisons post test). Data are shown as the mean ± SD. Source data are provided as a Source Data file.

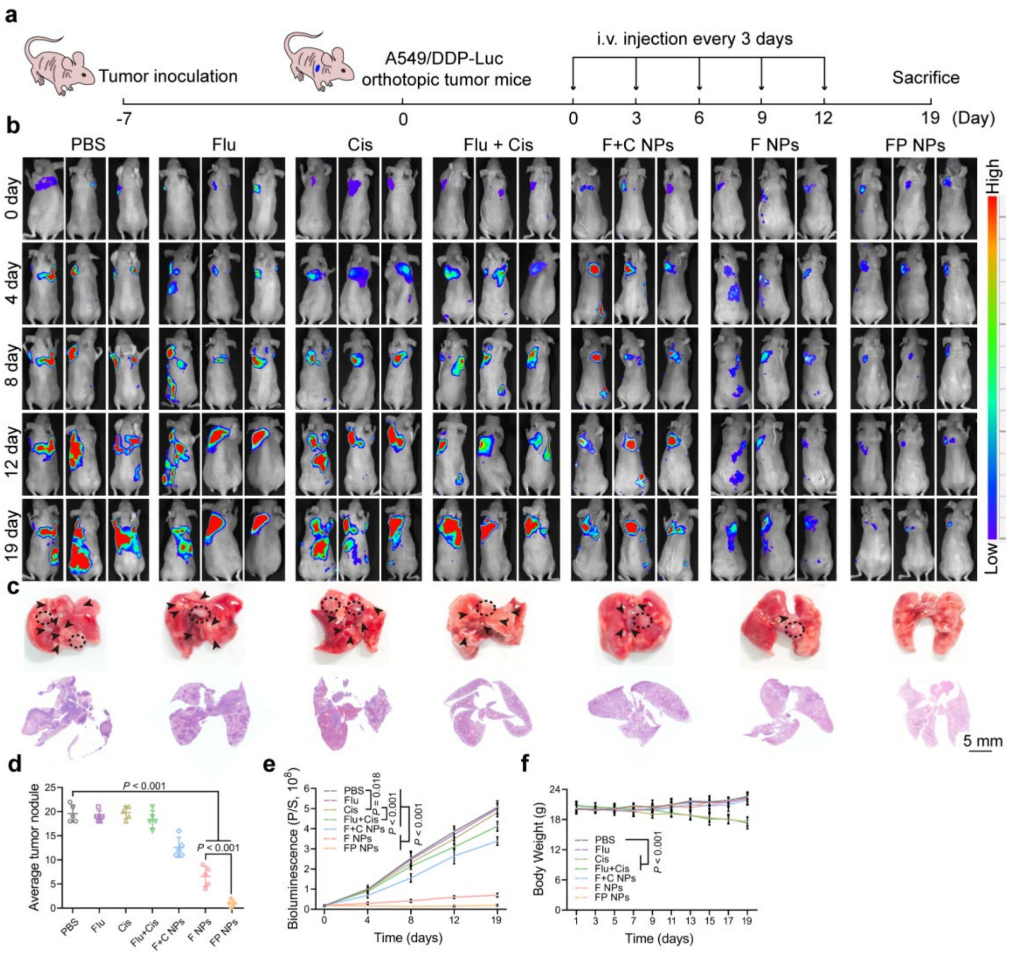

Supplementary Fig. 48 Antitumor efficacy of intravenously injected FP NPs in A549/DDP-luc orthotopic lung tumors. **a** Schematic illustration of the experimental design. **b** The progression of orthotopic lung tumors (n = 5 mice per group) in BALB/c nude mice. A representative bioluminescent image from each group is shown. **c** The final anatomical picture and the H&E staining of the lungs. Scale bars, 5 mm. **d** Average tumor nodule. Tumor nodules of 2-10 mm^3 in volume were counted using harvested lungs from the control and treated groups, and the average number of tumor nodules was determined. Each dot represents a tumor from an individual mouse. The tumor nodules in the lungs are indicated by arrows (one-way ANOVA followed by Tukey's HSD post hoc test). **e** Quantitative bioluminescence

analysis in mice bearing orthotopic lung tumors; P/S = photons/second (two-way ANOVA followed by Tukey’s multiple comparisons posttest). **f** Mouse body weight in the orthotopic lung tumor mouse model (two-way ANOVA followed by Tukey’s multiple comparisons posttest). Data are shown as the mean ± SD Source data are provided as a Source Data file.

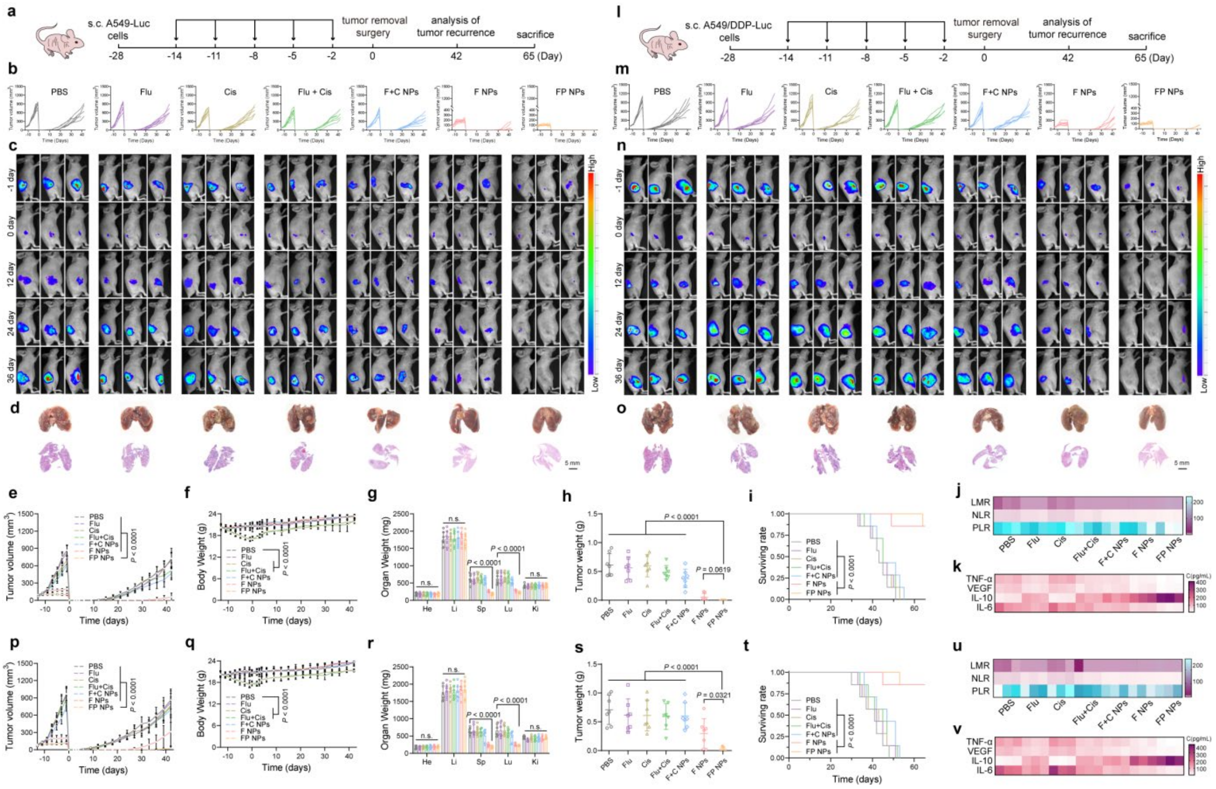

Supplementary Fig. 49 The tumor recurrence and metastasis inhibition of FP NPs in A549-luc and A549/DDP-luc tumor-bearing mice. a, **l** Schematic illustration of the experimental design. b, m Tumor volume changes for each mouse in each group (*n* = 7 mice per group). **c**, **n** In vivo bioluminescence imaging of tumor-bearing mice receiving various treatments after surgery. Three representative mice in each treatment group are shown. Images of day 0 were taken on the day of surgery. **d**, **o** The final anatomical picture and H&E staining of the lungs (*n* = 3 independent samples; Scale bars, 5 mm). **e**, **p** The tumor volumes of mice during treatments with each group (*n* = 7 mice per group; two-way ANOVA followed by Tukey’s multiple comparisons post test). **f**, **q** The body weight of mice during treatments with each group (*n* = 7 mice per group; two-way ANOVA followed by Tukey’s multiple comparisons post test). **g**, **r** Final weights of the heart, liver, spleen, lungs, and kidneys (*n* = 7 mice per group;two-tailed unpaired t test). **h**, **s** The recurrence tumor weights of different groups on day 42 after surgery (*n* = 7 mice per group; one-way ANOVA followed by Tukey’s HSD post hoc test). **i**, **t** Kaplan-Meier survival curve of mice treated with each group over 65 days (*n* = 7 mice per group; Log-rank Mantel–Cox test). **j**, **u** Heatmap of TNF-α, VEGF, IL-

10 and IL-6 expression profiles in serum (n = 3 mice per group). **k**, **v** Indicators of routine blood examination (n = 3 mice per group; two-tailed unpaired t test) of mice. Data are shown as the mean ± SD; n.s. = no significance. Source data are provided as a Source Data file.

Supplementary Table 1. Univariate and multivariate Cox regression analysis for overall survival in all patients. CI, confidence interval; HR, hazard ratio. Wald tests were performed to etermine significance.

Variable	Univariate analysis			Multivariate analysis		
Factor	HR	95% CI	*p*-value	HR	95% CI	*p*-value
Sex						
Male	1			1		
Female	0.741	0.600-0.916	0.006	0.898	0.720-1.120	0.339
Stage						
I	1			1		
II	1.575	1.210-2.048	0.001	1.594	1.223-2.079	0.001
III	2.135	1.654-2.754	0.000	2.276	1.758-2.947	0.000
IV	3.744	2.487-5.636	0.000	4.067	2.692-6.144	0.000
Person cigarette pack year value						
≤28	1			1		
> 28	1.333	1.085-1.637	0.006	1.183	0.949-1.476	0.135

Supplementary Table 2. Reverse sequencing analysis of the *TP53* gene in untreated A549 cells.

Different sequences	Sequence
Sequence 1(from 10730 to 11625, length=896 bp)	GGGTGGGGCAGGAGTGCTTGGGTTGTGGTGA AACATTGGAAGAGAGAATGTGAAGCAGCCAT TCTTTTCCTGCTCCACAGGAAGCCGAGCTGTC TCAGACACTGGCATGGTGTTGGGGGAGGGGG

	TTCCTTCTCTGCAGGCCCAGGTGACCCAGGGT TGGAAGTGTCTCATGCTGGATCCCCACTTTTC CTCTTGCAGCAGCCAGACTGCCTTCCGGGTCA CTGCCATGGAGGAGCCGCAGTCAGATCCTAG CGTCGAGCCCCCTCTGAGTCAGGAAACATTTT CAGACCTATGGAAACTGTGAGTGGATCCATT GGAAGGGCAGGCCCACCACCCCCACCCCAAC CCCAGCCCCCTAGCAGAGACCTGTGGGAAGC GAAAATTCCATGGGACTGACTTTCTGCTCTTG TCTTTCAGACTTCCTGAAAACAACGTTCTGGT AAGGACAAGGGTTGGGCTGGGGACCTGGAG GGCTGGGGACCTGGAGGGCTGGGGGGCTGGG GGGCTGAGGACCTGGTCCTCTGACTGCTCTTT TCACCCATCTACAGTCCCCCTTGCCGTCCCAA GCAATGGATGATTTGATGCTGTCCCCGGACG ATATTGAACAATGGTTCACTGAAGACCCAGG TCCAGATGAAGCTCCCAGAATGCCAGAGGCT GCTCCCCCCGTGGCCCCTGCACCAGCAGCTCC TACACCGGCGGCCCCTGCACCAGCCCCCTCCT GGCCCCTGTCATCTTCTGTCCCTTCCCAGAAA ACCTACCAGGGCAGCTACGGTTTCCGTCTGG GCTTCTTGCATTCTGGGACAGCCAAGTCTGTG ACTTGCACGGTCAGTTGCCCTGAGGGGCTGG CTTCCATGAGACTTCAATGCCTGGCCGTATCC CCCTGCATTTCTTT
Sequence 2(from 12227 to12746, length=520 bp)	TGTTTGTTTCTTTGCTGCCGTCTTCCAGTTGCT TTATCTGTTCACTTGTGCCCTGACTTTCAACT CTGTCTCCTTCCTCTTCCTACAGTACTCCCCTG CCCTCAACAAGATGTTTTGCCAACTGGCCAA GACCTGCCCTGTGCAGCTGTGGGTTGATTCCA CACCCCCGCCCGGCACCCGCGTCCGCGCCAT GGCCATCTACAAGCAGTCACAGCACATGACG GAGGTTGTGAGGCGCTGCCCCCACCATGAGC GCTGCTCAGATAGCGATGGTGAGCAGCTGGG GCTGGAGAGACGACAGGGCTGGTTGCCCAGG

	GTCCCCAGGCCTCTGATTCCTCACTGATTGCT<br>CTTAGGTCTGGCCCCTCCTCAGCATCTTATCC<br>GAGTGGAAGGAAATTTGCGTGTGGAGTATTT<br>GGATGACAGAAACACTTTTCGACATAGTGTG<br>GTGGTGCCCTATGAGCCGCCTGAGGTCTGGTT<br>TGCAACTGGGGTCTCTGGGAGGAGGGGTTAA<br>GGGTGGTTGTCAGTG
Sequence 3(from 13175 to 14252, length=1078 bp)	AAAAAAAAAAAAAAAAGGCCTCCCCTGCTTG<br>CCACAGGTCTCCCCAAGGCGCACTGGCCTCA<br>TCTTGGGCCTGTGTTATCTCCTAGGTTGGCTC<br>TGACTGTACCACCATCCACTACAACTACATGT<br>GTAACAGTTCCTGCATGGGCGGCATGAACCG<br>GAGGCCCATCCTCACCATCATCACACTGGAA<br>GACTCCAGGTCAGGAGCCACTTGCCACCCTG<br>CACACTGGCCTGCTGTGCCCCAGCCTCTGCTT<br>GCCTCTGACCCCTGGGCCCACCTCTTACCGAT<br>TTCTTCCATACTACTACCCATCCACCTCTCAT<br>CACATCCCCGGCGGGGAATCTCCTTACTGCTC<br>CCACTCAGTTTTCTTTTCTCTGGCTTTGGGAC<br>CTCTTAACCTGTGGCTTCTCCTCCACCTACCT<br>GGAGCTGGAGCTTAGGCTCCAGAAAGGACAA<br>GGGTGGTTGGGAGTAGATGGAGCCTGGTTTT<br>TTAAATGGGACAGGTAGGACCTGATTTCCTT<br>ACTGCCTCTTGCTTCTCTTTTCCTATCCTGAGT<br>AGTGGTAATCTACTGGGACGGAACAGCTTTG<br>AGGTGCGTGTTTGTGCCTGTCCTGGGAGAGA<br>CCGGCGCACAGAGGAAGAGAATCTCCGCAAG<br>AAAGGGGAGCCTCACCACGAGCTGCCCCCAG<br>GGAGCACTAAGCGAGGTAAGCAAGCAGGAC<br>AAGAAGCGGTGGAGGAGACCAAGGGTGCAG<br>TTATGCCTCAGATTCACTTTTATCACCTTTCCT<br>TGCCTCTTTCCTAGCACTGCCCAACAACACCA<br>GCTCCTCTCCCCAGCCAAAGAAGAAACCACT<br>GGATGGAGAATATTTCACCCTTCAGGTACTA<br>AGTCTTGGGACCTCTTATCAAGTGGAAAGTTT

	CCAGTCTAACACTCAAAATGCCGTTTTCTTCT<br>TGACTGTTTTACCTGCAATTGGGGCATTTGCC<br>ATCAGGGGGCAGTGATGCCTCAAAGACAATG<br>GCTCCTGGTTGTAGCTAACTAACTTCAGAACA<br>CCAACTTATACCATAATATATATTTTAAAGGA<br>CCAGACCAGCTTTCAAAAAGAAAATTGTTAA<br>AGAGAGCA
