# Supplementary material for: Nanoparticles targeting mutant p53 overcome chemoresistance and tumor recurrence in non-small cell lung cancer
Source: Nat Commun. 2024 Mar 29;15:2759. doi: 10.1038/s41467-024-47080-3 (PMC10980692; doi:10.1038/s41467-024-47080-3)
Supplement: Supplementary file 2 — Reporting Summary [file 41467_2024_47080_MOESM2_ESM.pdf]

nature portfolio

Corresponding author(s): Hu-Lin Jiang

Last updated by author(s): Mar 5, 2024

Reporting Summary

Nature Portfolio wishes to improve the reproducibility of the work that we publish. This form provides structure for consistency and transparency in reporting. For further information on Nature Portfolio policies, see our Editorial Policies and the Editorial Policy Checklist.

Statistics

For all statistical analyses, confirm that the following items are present in the figure legend, table legend, main text, or Methods section.

n/a	Confirmed	
☐	☒	The exact sample size (*n*) for each experimental group/condition, given as a discrete number and unit of measurement
☐	☒	A statement on whether measurements were taken from distinct samples or whether the same sample was measured repeatedly
☐	☒	The statistical test(s) used AND whether they are one- or two-sided<br>*Only common tests should be described solely by name; describe more complex techniques in the Methods section.*
☐	☒	A description of all covariates tested
☐	☒	A description of any assumptions or corrections, such as tests of normality and adjustment for multiple comparisons
☐	☒	A full description of the statistical parameters including central tendency (e.g. means) or other basic estimates (e.g. regression coefficient) AND variation (e.g. standard deviation) or associated estimates of uncertainty (e.g. confidence intervals)
☐	☒	For null hypothesis testing, the test statistic (e.g. F, t, r) with confidence intervals, effect sizes, degrees of freedom and P value noted<br>*Give P values as exact values whenever suitable.*
☒	☐	For Bayesian analysis, information on the choice of priors and Markov chain Monte Carlo settings
☒	☐	For hierarchical and complex designs, identification of the appropriate level for tests and full reporting of outcomes
☒	☐	Estimates of effect sizes (e.g. Cohen's *d*, Pearson's *r*), indicating how they were calculated

Our web collection on statistics for biologists contains articles on many of the points above.

Software and code

Policy information about availability of computer code

Data collection	No custom-made code was required to collect data.<br>(1) The H&E staining images were collected using digital slice scanning device (NanoZoomer 2.0 RS, Hamamatsu, Japan).<br>(2) The immunofluorescence images were collected using digital slice scanning device (Pannoramic MIDI/250 ,3D HISTECH, Hungary).<br>(3) The fluorescence images were collected using confocal laser scanning microscopy (Carl ZEISS 700, ZEISS, Germany) and inverted fluorescence microscope (Ti-s, Nikon, Japan).<br>(4) The morphology of MLPers was monitored by scanning electron microscope (HITACHI Regulus 8100, HITACHI, Japan).<br>(5) The Western blot images were detected by the chemiluminescence imaging system (Tanon 4200, Tanon, China).<br>(6) The MFI of MitoTracker Green was detected by flow cytometry (BD FACSCalibur, BD, USA).<br>(7) The uv absorbance was monitored by microplate reader (Multiskan Go, Thermo Fisher, USA).<br>(8) The automatic animal blood cell analyzer (BC-2800Vet, Mai Rui, China) is used for the detection of blood routine test.<br>(9) The average hydrodynamic diameters and zeta potentials of Mito and MLPers were monitored by dynamic light scattering (DLS) and Nano ZS zetasizer of DLS analyzer (Brookhaven, Germany).
Data analysis	Graphpad Prism v8 was used for general statistical analysis, ImageJ vl.52 for image processing, FlowJo 7.6.1 for flow cytometry data analysis and SPSS 19.0 for data statistics.

For manuscripts utilizing custom algorithms or software that are central to the research but not yet described in published literature, software must be made available to editors and reviewers. We strongly encourage code deposition in a community repository (e.g. GitHub). See the Nature Portfolio guidelines for submitting code & software for further information.

Data

Policy information about availability of data

All manuscripts must include a data availability statement. This statement should provide the following information, where applicable:

- Accession codes, unique identifiers, or web links for publicly available datasets
- A description of any restrictions on data availability
- For clinical datasets or third party data, please ensure that the statement adheres to our policy

The RNA sequencing data used in this study are available at National Center for Biotechnology Information Sequence Read Archive (SRA) database with accession code PRJNA1047938. The authors declare that all other data supporting the findings of this study are available within the article and its Supplementary Information files. Should any data files be needed in another format they are available from the corresponding author upon reasonable request. Source data are provided with this paper.

Human research participants

Policy information about studies involving human research participants and Sex and Gender in Research.

Reporting on sex and gender	*Use the terms sex (biological attribute) and gender (shaped by social and cultural circumstances) carefully in order to avoid confusing both terms. Indicate if findings apply to only one sex or gender; describe whether sex and gender were considered in study design whether sex and/or gender was determined based on self-reporting or assigned and methods used. Provide in the source data disaggregated sex and gender data where this information has been collected, and consent has been obtained for sharing of individual-level data; provide overall numbers in this Reporting Summary. Please state if this information has not been collected. Report sex- and gender-based analyses where performed, justify reasons for lack of sex- and gender-based analysis.*
Population characteristics	*Describe the covariate-relevant population characteristics of the human research participants (e.g. age, genotypic information, past and current diagnosis and treatment categories). If you filled out the behavioural & social sciences study design questions and have nothing to add here, write "See above."*
Recruitment	*Describe how participants were recruited. Outline any potential self-selection bias or other biases that may be present and how these are likely to impact results.*
Ethics oversight	*Identify the organization(s) that approved the study protocol.*

Note that full information on the approval of the study protocol must also be provided in the manuscript.

Field-specific reporting

Please select the one below that is the best fit for your research. If you are not sure, read the appropriate sections before making your selection.

- [x] Life sciences
- [ ] Behavioural & social sciences
- [ ] Ecological, evolutionary & environmental sciences

For a reference copy of the document with all sections, see nature.com/documents/nr-reporting-summary-flat.pdf

Life sciences study design

All studies must disclose on these points even when the disclosure is negative.

Sample size	The sample sizes of this study were determined on the basis of similar published studies (Liang, et al. Nat Biomed Eng 2019, 3(9): 729-740). In antitumor experiments, 5-8 mice each group were used to analyze tumor volume, tumor weight, and the survival rate. For other experiments, the sample size for each group was 3-5.
Data exclusions	No data were excluded in the analysis.
Replication	Experiments were independently repeated as indicated and experimental findings were reproducible (described in figure legends for further details).
Randomization	All samples were randomly allocated into experimental groups.
Blinding	Investigators were blinded during tumor inoculation, animal grouping and animal drug treatment. Survival data were determined by blinded staff. However, investigators were not blinded to collect data of tumor volume and weight, because the same investigator processed the animals and analyzed the data. For in vitro experiments, blinding was impossible because a single investigator processed all steps of the experiment, and there was no step that introduced personal bias.

Reporting for specific materials, systems and methods

We require information from authors about some types of materials, experimental systems and methods used in many studies. Here, indicate whether each material, system or method listed is relevant to your study. If you are not sure if a list item applies to your research, read the appropriate section before selecting a response.

Materials & experimental systems

n/a	Involved in the study
☐	☒ Antibodies
☐	☒ Eukaryotic cell lines
☒	☐ Palaeontology and archaeology
☐	☒ Animals and other organisms
☒	☐ Clinical data
☒	☐ Dual use research of concern

Methods

n/a	Involved in the study
☒	☐ ChIP-seq
☐	☒ Flow cytometry
☒	☐ MRI-based neuroimaging

Antibodies

Antibodies used

anti-p53 (Santa Cruz Biotechnology, sc-126; 1:1,000 dilution), anti-VEGF (Santa Cruz Biotechnology, sc-7269; 1:1,000 dilution), anti-eIF2α (Cell Signaling Technology, #5324; 1:1,000 dilution), anti-Phospho-eIF2α (Cell Signaling Technology, #3398; 1:1,000 dilution), anti-Phospho-H2A.X (Servicebio, GB11181; 1:1,000 dilution), anti-MMP2 (Servicebio, GB11130; 1:1,000 dilution), anti-MMP9 (Servicebio, GB11132; 1:1,000 dilution), anti-E-cadherin (Servicebio, GB12083; 1:1,000 dilution), anti-Vimentin (Servicebio, GB111308; 1:1,000 dilution), anti-ATF4 (Beyotime, AF2560; 1:1,000 dilution), anti-DDIT3/CHOP (Beyotime, AF6684; 1:1,000 dilution).

Validation

All vendors showed validation data for antibodies on their websites (including Western blot, IF etc.)
Manufacturer's website:
anti-p53 (https://www.scbt.com/p/p53-antibody-do-1), anti-VEGF (https://www.scbt.com/zh/p/vegf-antibody-c-1), anti-eIF2α (https://www.cellsignal.cn/products/primary-antibodies/eif2a-d7d3-xp-rabbit-mab/5324), anti-Phospho-eIF2α (https://www.cellsignal.com/products/primary-antibodies/phospho-eif2a-ser51-d9g8-xp-rabbit-mab/3398), anti-Phospho-H2A.X (https://www.servicebio.cn/goodsdetail?id=4702), anti-MMP2 (https://www.servicebio.cn/goodsdetail?id=4702), anti-MMP9 (https://www.servicebio.cn/goodsdetail?id=1401), anti-E-cadherin (https://www.servicebio.cn/goodsdetail?id=4869), anti-Vimentin (https://www.servicebio.cn/goodsdetail?id=3663), anti-ATF4 (https://beyotime.com/product/AF2560.htm), anti-DDIT3/CHOP (https://www.beyotime.com/product/AF6684.htm).

Eukaryotic cell lines

Policy information about cell lines and Sex and Gender in Research

Cell line source(s)

The p53 mutant human NSCLC cell lines NCI-H1975 (American Type Culture Collection (ATCC), #CRL-5908), NCI-H2087 (ATCC, #CRL-5922), NCI-H2342 (ATCC, #CRL-5941), NCI-H1793 (ATCC, #CRL-5896), NCI-H2196 (ATCC, #CRL-5932), NCI-H748 (ATCC, #CRL-5841), NCI-H1770 (ATCC, #CRL-5893), NCI-H1591 (ATCC, #CRL-5878), NCI-H520 (ATCC, #HTB-182) were purchased from ATCC. The p53 null human NSCLC cell lines NCI-H1299 (ATCC, #CRL-5803), was purchased from ATCC. The p53 wild-type human NSCLC cell line A549 (ATCC, #CCL-185) was purchased from ATCC. The cisplatin-resistance human NSCLC cell line A549/DDP (Procell, #CL-0519) was purchased from Cell Resource Center (Procell Life Science & Technology Co., Ltd., China), obtained from cisplatin-induced A549 cells. The cell line NCI-H1975-luc (Aoyinbio, SAc0273LG), A549-luc (Aoyinbio, SAc0023LG), A549/DDP-luc (Aoyinbio, SAc0024LG) was purchased from Cell Resource Center (Aoyinbio Co., Ltd., China). The A549/DDP cell culture medium was supplemented with 1% penicillin/streptomycin (Thermo-Fisher Scientific), 10% fetal bovine serum (FBS; Gibco) and 1-2 µg/mL cisplatin. The other cell culture medium was supplemented with 1% penicillin/streptomycin (Thermo-Fisher Scientific) and 10% fetal bovine serum (FBS; Gibco).

Authentication

Authentication via STR profiling was performed by the commercial suppliers before purchase of the material. We also retested before the experiment was performed.

Mycoplasma contamination

All cell lines are tested in a regulatory basis (every 3 months) to rule out any mycoplasma contamination, all cells tested negative for mycoplasma contamination.

Commonly misidentified lines
(See ICLAC register)

No commonly misidentified cell lines were used.

Animals and other research organisms

Policy information about studies involving animals; ARRIVE guidelines recommended for reporting animal research, and Sex and Gender in Research

Laboratory animals

Female and male BALB/c nude mice, four- to five-week-old, were purchased from Changzhou Cavens Laboratory Animal Co., Ltd. (Jiangsu, China).
Subcutaneous tumor model: The mice housing in SPF environment were subcutaneously injected with 100 µL H1975 cells (1.0×10^7

per mL). When the tumors grew to about 100 mm^3, the mice were randomly divided according to the experimental needs. Orthotopic lung cancer model: Cells were labeled with luciferase and mixed 1:1 with matrix gel to a final concentration of 1.0×10^7/mL. Mice were anesthetized with isoflurane (flow rate of 500/1000 mL/min), with the vaporizer set at 5%, until signs of recumbency were present. Mice were placed in the right lateral recumbent position, 1 cm incision was made after disinfection. 1 mL syringe was used to puncture vertically at the upper edge of the ribs for about 3 mm, slowly injecting about 50 μL of cell suspension staying for 3 to 5 seconds, quickly withdrawing the needle, disinfecting, and suturing with iodine povidone. The wounds could be healed in 4 to 6 days, so as to establish the orthotopic grafted tumor model of lung cancer.

Wild animals: No wild animals were used in this study.

Reporting on sex: Balb/c female mice is often used for tumor research. In order to avoid the biases associated with sex differences in the immune system and tumor growth, only females were selected in the main experiment.

Field-collected samples: This study did not involve samples collected from the field.

Ethics oversight: All the animal protocols and procedures were performed under the guidelines for human and responsible use of animals in research approved by the regional ethics committee of China Pharmaceutical University (2022-08-009).

Note that full information on the approval of the study protocol must also be provided in the manuscript.

Flow Cytometry

Plots

Confirm that:

- [x] The axis labels state the marker and fluorochrome used (e.g. CD4-FITC).
- [x] The axis scales are clearly visible. Include numbers along axes only for bottom left plot of group (a 'group' is an analysis of identical markers).
- [ ] All plots are contour plots with outliers or pseudocolor plots.
- [x] A numerical value for number of cells or percentage (with statistics) is provided.

Methodology

Sample preparation: 3×10^5 H1975 cells were seeded into 6-well plates. After attachment overnight, cells were treated with cisplatin, fluvastatin sodium, mixture of dual drugs and FP NPs, then all the attached cells were trypsinized and collected to disperse in 1× binding buffer solution (ice-cold) at a concentration of 3×10^5 cells. Then cells were incubated the mixed cell suspension of FITC Annexin V and PI at room temperature for 20 min in darkness and performed analysis by flow cytometry.

Instrument: Flow cytometry (BD FACSCalibur, USA).

Software: FlowJo 7.6.1

Cell population abundance: No cell sorting was performed.

Gating strategy: Cells were discriminated from debris and clumps using FSC-A/SSC-A gating strategy based on experience. Cell populations within the gate were further analysed based on the expression of markers. The gating strategy is provide in Supplementary Fig.29.

- [x] Tick this box to confirm that a figure exemplifying the gating strategy is provided in the Supplementary Information.
